# Divergent impacts of estradiol/testosterone reduction on biological aging: optimal HRT window in females recommended

**DOI:** 10.1186/s13293-026-00873-1

**Published:** 2026-03-13

**Authors:** Yicheng Ma, Junming Han, Qihang Li, Xiao Jiang, Yuan Li, Keke Zhang, Ling Gao

**Affiliations:** 1 Key Laboratory of Endocrine Glucose & Lipids Metabolism and Brain Aging, Ministry of Education, Department of Endocrinology, Shandong Provincial Hospital Affiliated to Shandong First Medical University, Jinan, 250021 Shandong China; 2 Shandong Clinical Research Center of Diabetes and Metabolic Diseases, Jinan, 250021 Shandong China; 3 Shandong Institute of Endocrine and Metabolic Diseases, Jinan, 250021 Shandong China; 4 “Chuangxin China” Innovation Base of Stem Cell and Gene Therapy for Endocrine Metabolic Diseases, Jinan, China; 5 Shandong Engineering Laboratory of Prevention and Control for Endocrine and Metabolic Diseases, Jinan, 250021 Shandong China; 6 Shandong Engineering Research Center of Stem Cell and Gene Therapy for Endocrine and Metabolic Diseases, Jinan, 250021 Shandong China; 7 Department of General Medicine, Shandong Provincial Hospital Affiliated to Shandong First Medical University, Jinan, China

**Keywords:** Estradiol, Testosterone, Biological age acceleration, Hormone replacement therapy

## Abstract

**Background:**

The age-specific magnitude of the impact of hormones on aging and the role of hormone replacement therapy (HRT) remain unclear.

**Methods:**

We used data from UK Biobank participants for our analysis. Linear and logistic regression models were employed to investigate the associations and its differences between estradiol/testosterone and biological age acceleration (BAA) across different age group. Then, females were divided into HRT and non-HRT groups, and restricted cubic splines were used to evaluate HRT initiation age and duration.

**Results:**

A total of 54,912 participants (71.99% females, 28.01% males) were enrolled, with 39,303 females assessed for the impact of HRT on aging. Estradiol decline in females was linked to a 0.18—0.29-year BAA increase, while testosterone decline in males was linked to a 0.07—0.71-year BAA increase. It showed estradiol protected against aging in females (most prominent at 41–55 years) while elevated testosterone accelerated aging. In males, testosterone was protective (most significant at 56–70 years) and estradiol had no notable effect. Quartile and age-stratified analyses confirmed these findings. In contrast, the decrease of estradiol was associated with biological age younger. Hormone replacement therapy (HRT) in females resulted in sustained BAA reduction (-4.5 to -6.0, 41–70 years) superior to non-HRT (-4.0 to -5.5). HRT initiated at 56–60 years showed optimal efficacy, with longer duration associated with more pronounced aging deceleration.

**Conclusions:**

The impact of per standard deviation (SD) decrease in sex hormone levels on aging (BAA) varies by age, with significant effects observed in females aged 50–55 years and males aged 65–70 years. These findings may facilitate the optimization of HRT timing to maximize anti-aging benefits and enable personalized treatment strategies.

**Supplementary Information:**

The online version contains supplementary material available at 10.1186/s13293-026-00873-1.

## Abstract (plain language summary)

Aging and the decline in sex hormone levels are both nonlinear processes. It is well established that hormonal decline impacts aging. However, the age-specific magnitude of this effect and the role of hormone replacement therapy (HRT) remain unclear. We addressed it by examining related trends and impacts using the UK Biobank database. Our findings indicate that, as individuals age, the rate of hormone decline varies, and its effect on aging differs accordingly. In females, the decrease in estrogen levels accelerates aging, while the decrease in testosterone appears to have a protective effect. The most significant impact is observed in females aged 50-55 years. In males, the decline in testosterone exacerbates aging, with the most pronounced effects occurring in males aged 65-70 years. In females who underwent HRT, they had a younger biological age compared to those who did not. Initiating HRT in the 56-60 years age group produced the greatest benefits in decelerating aging, with a dose-dependent response.

**Supplementary Information:**

The online version contains supplementary material available at 10.1186/s13293-026-00873-1.

## Highlights

1. The impact of per SD decrease in sex hormone levels on aging varies by age.
2. Declines in sex hormone levels are associated with BAA, with the strongest effects observed in females aged 50—55 years and males aged 65—70 years.
3. Hormone replacement therapy (HRT) in females was associated with reductions in BAA, with optimal benefits observed when initiated between 56—60 years of age.
4. Prolonged HRT duration further enhanced its anti-aging effects in females.

**Supplementary Information:**

The online version contains supplementary material available at 10.1186/s13293-026-00873-1.

## Introduction

Aging is characterized by a progressive decline in physiological function and adaptive resilience across multiple organ systems. It initiates with cellular-level perturbations that enhance the vulnerability of tissues and organs. Over time, these cumulative changes precipitate the onset of disease, functional impairment, and ultimately, mortality. [1], [2] Biological age quantifies an individual’s physiological state and can be estimated using methodologies such as Klemera and Doubal’s method (KDM), PhenoAge, and GrimAge. Among these methodologies, KDM exhibits particular efficacy in predicting age-related health outcomes. [3], [4] The discrepancy between biological and chronological age, referred to Biological Age Acceleration (BAA), indicates whether an individual is biologically younger or older than expected.

Multiple factors contribute to the acceleration of biological age. Psychological factors, such as unhappiness or loneliness, can increase biological age by up to 1.65 years. [5] Elevated mid-life serum sodium levels (> 142 mmol/L) [6] and the combination of short sleep duration with high physical activity [7] are also associated with older biological age. In contrast, dietary restrictions and reduced insulin-IGF signaling have been found to reverse numerous age-related changes, [8] while regular physical activity has been documented to decelerate aging. [9].

Sex hormone is another key factor influencing BAA. In females, the abrupt decline in estrogen levels after menopause increases the risk of osteoporosis, cardiovascular diseases, and metabolic disorders. [10] In males, gradual decline in testosterone contributes to muscle loss, fat accumulation, and cognitive decline. [11] Evidence further suggests that sex hormone levels influence biological aging: estrogen supplementation in postmenopausal females is associated with a younger biological age, [12] while higher testosterone levels and an elevated testosterone-to-estradiol ratio in males are associated with slower epigenetic aging. [13] Estradiol loss accelerates skin and skeletal aging in females, [14–16] whereas low testosterone levels in males is associated with increased all-cause mortality. [17] Consequently, these findings indicate that fluctuations in sex hormones substantially affect the rate of aging, as reflected by BAA. Importantly, aging is a nonlinear process. [18, 19] This indicates that the body’s response to both external stimuli and internal changes is age-specific. Thus, the impact of a unit decline in hormone levels varies across different ages due to age-related differences in systemic states such as immune and metabolic function. This variation manifests as the differences in the pace of aging, resulting in a discrepancy between biological and chronological age (BAA).

This study aims to estimate whether the effects of sex hormone decline on aging are uniform; if not, we seek to quantify the impact of sex hormone reduction on ageing across different age groups. The research aims to provide evidence to facilitate the optimization of hormone replacement therapy (HRT) interventions.

## Methods

### Study design and participants

This study utilized data from the UK Biobank (Application Number: 89483). The UK Biobank is a prospective cohort study that enrolled over 500,000 participants. These participants, aged 39–70 years, were recruited from across the United Kingdom and were enrolled between 2006 and 2010. [20] (http://www.ukbiobank.ac.uk) At baseline, participants provided sociodemographic, lifestyle, environmental, and health-related information via touchscreen questionnaires and verbal interviews. They also underwent physical examinations and provided biological samples (blood, urine, saliva) for genotyping and biochemical analyses. All participants provided written informed consent, and the study was approved by the North West Multicenter Research Ethics Committee.

This study analyzed 54,912 participants aged 41–70 years. Participants were excluded if they met any of the following criteria: [1] missing biomarkers required for biological age calculation, including estradiol, testosterone, glycated hemoglobin (HbA1c), total cholesterol (TC), albumin, alkaline phosphatase, creatine, urea, C-reactive protein (CRP), body mass index (BMI), and sex hormone-binding globulin (SHBG); [2] a history of hysterectomy or oophorectomy; [3] inability to compute biological age; [4] undetermined hormone replacement therapy (HRT) status. After applying the inclusion and exclusion criteria, a total of 54,912 participants were included in the present study and a detailed participant flow diagram was provided. (eFigure 1).

### Biological age and biological age acceleration

Biological age and BAA were calculated using the R package "BioAge", [2] which implements the KDM. [21] KDM biological age (KDMage) incorporates forced expiratory volume, systolic blood pressure, and seven hematological parameters: alkaline phosphatase, TC, albumin, creatinine, blood urea nitrogen, CRP, and HbA1c. BAA was calculated as the residual from a linear regression of BA against chronological age. [22] The variables used to calculate biological age, along with additional analytical items, are presented in eTable 1. Participants with BAA ≥ 0 were classified as having “aging acceleration”, and those with BAA < 0 were classified as having “aging deceleration”.

### Assay of sex hormones

Serum estradiol and testosterone were measured using a competitive immunoassay platform (Beckman Coulter DXI 800) by Chemiluminescent Immunoassay- competitive binding method. Detailed assay procedures and quality control protocols have been described previously by the UK Biobank. The lower limits of detection were 73 pmol/L for estradiol and 0.35 nmol/L for testosterone. (https://biobank.ndph.ox.ac.uk/showcase/refer.cgi?id=5636) (https://biobank.ndph.ox.ac.uk/showcase/refer.cgi?id=1227).

### Statistical analysis

Continuous variables (baseline age, systolic blood pressure, BMI, and laboratory parameters) were presented as medians with interquartile ranges (IQRs). Categorical variables were reported as counts and percentages (n, %). Group comparisons were performed using Kruskal–Wallis H tests for continuous variables, while chi-square tests were used for categorical variables. Post-hoc analyses were adjusted using the Benjamini–Hochberg correction.

Testosterone and estradiol levels were standardized. Linear regression was used to assess the correlation and significance between hormone levels and BAA. Generalized linear models were employed to estimate odds ratios (ORs) and 95% confidence intervals (CIs), which evaluated "aging acceleration" per standard deviation (SD) increase in sex hormone levels. The analyses were adjusted for factors including smoking status, alcohol use, and BMI. Restricted cubic splines were fitted to logistic regression models to evaluate how HRT initiation age and duration affected BAA.

Statistical significance was set at two-tailed P < 0.05. All analyses were conducted using R software (version 4.3.3; https://www.r-project.org/).

### Sensitivity analysis

The sensitivity analysis was performed using the Gompertz Law-based biological age (GOLD BioAge) method, which is derived from the Gompertz mortality law. To enhance computational efficiency and maintain consistency with the main analysis, we utilized a lightweight version (Light BioAge and Light BioAgeDiff). This version requires only three standard indicators, including creatinine, glucose, and C-reactive protein, yet explains 93.73% of the variation captured by the full version. [23].

## Results

### Baseline characteristics of the study population

A total of 54,912 people were included in this study population, comprising 39,529 females (71.99%) and 15,383 males (28.01%). The majority (75.9%) of females were aged between 41 and 50 years, in contrast to a more evenly distributed age range in males. (eFigure 2) The majority of participants were non-smokers but reported alcohol consumption. Among female participants, the average chronological age was 47.00 years [IQR: 44.00, 51.00], with an average biological age of 41.41 years [IQR: 38.07, 45.48], resulting in an average BAA of -5.47 years [IQR: -7.43, -3.44]. In female participants, the average estradiol and testosterone levels were 407.80 pmol/L [IQR: 271.30, 653.80] and 1.15 nmol/L [IQR: 0.84, 1.52], respectively. Among male participants, the average chronological age was 58.00 years [IQR: 50.00, 64.00], with an average biological age of 48.94 years [IQR: 41.29, 56.44], leading to an average BAA of -8.21 years [IQR: -13.00, -3.09]. The average estradiol and testosterone in man were 204.10 pmol/L [IQR: 188.80, 230.90] and 12.89 nmol/L [IQR: 10.51, 15.69], respectively. (Table 1).

**Table 1:** Baseline characteristics of participants by gender

Variables ^a^	Female (n = 39,529)	Male (n = 15,383)	P value ^b^
Age, y	47.00 [44.00, 50.00]	58.00 [50.00, 64.00]	< 0.001
Age Group, y, n (%)			< 0.001
41–45	15,847 (40.1)	1775 (11.5)	
46–50	14,134 (35.8)	2133 (13.9)	
51–55	6469 (16.4)	2382 (15.5)	
56–60	1670 (4.2)	2857 (18.6)	
61–65	964 (2.4)	3621 (23.5)	
66–70	445 (1.1)	2615 (17.0)	
Smoking, n (%)			< 0.001
prefer not to answer	104 (0.3)	66 (0.4)	
never	24,891 (63.1)	7583 (49.3)	
previous	10,500 (26.6)	5775 (37.6)	
current	3981 (10.1)	1948 (12.7)	
Drinking, n (%)			< 0.001
prefer not to answer	44 (0.1)	29 (0.2)	
never	1753 (4.4)	443 (2.9)	
previous	1100 (2.8)	582 (3.8)	
current	36,579 (92.7)	14,318 (93.1)	
SBP, mm Hg	124.50 [114.50, 136.00]	139.00 [127.50, 151.50]	< 0.001
BMI, kg/m2	25.49 [22.94, 29.26]	27.70 [25.23, 30.73]	< 0.001
Waist circumference, cm	80.00 [74.00, 90.00]	97.00 [90.00, 105.00]	< 0.001
Hip circumference, cm	101.00 [96.00, 108.00]	103.00 [99.00, 109.00]	< 0.001
Glucose, mmol/L	4.77 [4.48, 5.10]	4.94 [4.60, 5.35]	< 0.001
HbA1c, mmol/L	33.20 [31.00, 35.50]	35.30 [32.70, 38.30]	< 0.001
HDL-c, mmol/L	1.52 [1.30, 1.77]	1.24 [1.06, 1.45]	< 0.001
LDL-c, mmol/L	3.26 [2.79, 3.78]	3.37 [2.79, 3.95]	< 0.001
TG, mmol/L	1.09 [0.81, 1.54]	1.54 [1.09, 2.20]	< 0.001
Cholesterol, mmol/L	5.37 [4.77, 6.03]	5.32 [4.59, 6.08]	< 0.001
Testosterone, nmol/L	1.15 [0.84, 1.52]	12.89 [10.51, 15.69]	< 0.001
Estradiol, pmol/L	407.80 [271.30, 653.80]	204.10 [188.80, 230.90]	< 0.001
Biological age, y	41.41 [38.07, 45.48]	48.94 [41.29, 56.44]	< 0.001
Biological age acceleration, y	-5.47 [-7.43, -3.44]	-8.21 [-13.00, -3.09]	< 0.001

^a^ The description of continuous variables is represented by median and interquartile range, while the description of categorical variables is represented by n (%)

^b^ Group differences were compared using Student t test, χ2 test, or Wilcoxon rank test

SBP, Systolic blood pressure; BMI, Body Mass Index; HbA1c, glycated haemoglobin A1c; HDL-c, High density lipoprotein cholesterol; LDL-c, High density lipoprotein cholesterol; TG, Triglycerides

### Sex-stratified association analysis of sex hormone levels with BAA

Through depicting the trends of BAA and sex hormone levels across ages using smoothing curves, we found that both BAA and sex hormone levels varied with aging and the changes exhibited a certain correlation. (eFigure 3, eFigure 4) Consequently, we conducted linear regression analyses on BAA, estradiol, and testosterone levels stratified by sex. After standardizing hormone levels, we found that the effect of per SD decrease in sex hormone levels on aging differed across age groups. Estradiol exerted a protective effect against aging in females. Specifically, per SD decrease in the standardized estradiol was associated with biological age older 0.18 (95%CI: 0.14–0.22), 0.20 (95%CI: 0.15–0.25), 0.29 (95%CI: 0.22–0.37), 0.04 (95%CI: -0.13–0.21), 0.04 (95%CI: -0.18–0.27) and 0.27 (95%CI: -0.11- 0.66) years in the corresponding 41–45, 46–50, 51–55, 56–60, 61–65 and 66–70 years of age groups, respectively. The association was robust in 41–45, 46–50, and 51–55 groups (adjusted p < 0.001). Conversely, estradiol did not show a significant effect on BAA in men. Testosterone, however, showed a significant protective effect in men. Specifically, per SD decrease in standardized testosterone was associated with biological age older

0.19 (95%CI: -0.14–0.52), 0.47 (95%CI: 0.14–0.80), 0.07 (95%CI: -0.27–0.41), 0.66 (95%CI: 0.33–0.99), 0.55 (95%CI: 0.25–0.85) and 0.71 (95%CI: 0.35–1.07) years in the corresponding 41–45, 46–50, 51–55, 56–60, 61–65 and 66–70 years of age groups, respectively. The association was robust in 56–60, 61–65 and 66–70 years of age groups (adjusted p < 0.001). In contrast, higher testosterone levels were linked to older age in females, per SD decrease in testosterone was associated with biological age younger 0.10 (95%CI: 0.05-0.14), 0.06 (95%CI: 0.01–0.11), 0.09 (95%CI: 0.01–0.17), 0.02 (95%CI: -0.15–0.18), 0.12 (95%CI: -0.10–0.34), and 0.10 (-0.28 - 0.48) years in the corresponding 41–45, 46–50, 51–55, 56–60, 61–65 and 66–70 years of age groups. (Table 2). These findings indicate that the roles of testosterone and estradiol in BAA differ significantly between sexes and evolve with age. Overall, fluctuations in sex hormone levels most notably influence BAA in females aged 51–55 years and men aged 66–70 years. Regarding females, we further hypothesize that aging is influenced by both the decline in estrogen levels and the increase in testosterone levels, with the reduction in estrogen levels being the predominant factor (β (estradiol): -0.18 to -0.29; β (testosterone): 0.06 to 0.010) (Table 2).

**Table 2:** Association of estradiol and testosterone reduction with biological age acceleration in different age groups

Gender	Age group, y	Estradiol, pmol/L			Testosterone, nmol/L		
		Est. ^a^	P	P. adj ^b^	Est	P	P. adj
**Female**	41–45	0.18 (0.14—0.22)	< 0.001	< 0.001	-0.10 (-0.14—-0.05)—	<0.001	<0.001
	46–50	0.20 (0.15—0.25)	< 0.001	< 0.001	-0.06 (-0.11—-0.01)	0.01	0.03
	51–55	0.29 (0.22—0.37)	< 0.001	< 0.001	-0.09 (-0.17—-0.01)	0.02	0.04
	56–60	0.04 (-0.13—0.21)	0.62	0.72	-0.02 (-0.18—0.15)	0.84	0.87
	61–65	0.04 (-0.18—0.27)	0.69	0.72	-0.12 (-0.34—0.10)	0.29	0.41
	66–70	0.27 (-0.11—0.66)	0.16	0.24	-0.10 (-0.48—0.28)	0.61	0.71
**Male**	41–45	-0.21 (-0.53—0.10)	0.19	0.26	0.19 (-0.14—0.52)	0.25	0.31
	46–50	0.17 (-0.14—0.49)	0.28	0.36	0.47 (0.14—0.80)	0.01	0.01
	51–55	-0.31 (-0.63—0.01)	0.06	0.10	0.07 (-0.27—0.41)	0.68	0.70
	56–60	0.03 (-0.28—0.34)	0.84	0.84	0.66 (0.33—0.99)	< 0.001	< 0.001
	61–65	-0.03 (-0.32—0.25)	0.82	0.84	0.55 (0.25—0.85)	< 0.001	< 0.001
	66–70	-0.36 (-0.71—-0.01)	0.04	0.08	0.71 (0.35—1.07)	< 0.001	< 0.001

^a^ The correlation and p-values between per SD sex hormone levels reduction and Biological Age Acceleration were determined using linear regression analysis. Est. was presented using the coefficient (β) and 95% confidence interval (95%CI)

^b^ P. adj Adjusted factors include smoking status, alcohol consumption status BMI

**Fig. 1:**
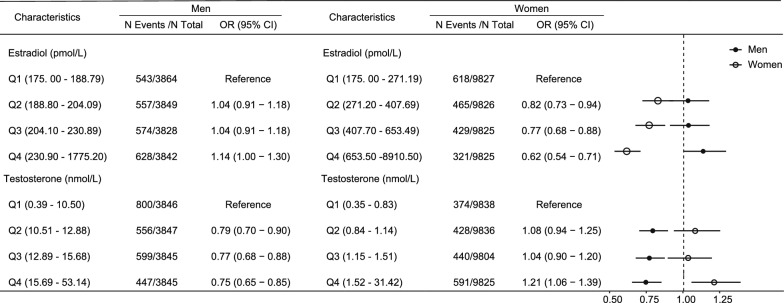
OR (95% CI) of aging acceleration events by sex-specific hormone quartiles. OR (95% CI) values were adjusted for smoking status, alcohol consumption, and BMI. Abbreviations: OR, odds ratio; CI, confidence interval; BMI, body mass index; aging acceleration, biological age acceleration ≥ 0; Q1, Q2, Q3, and Q4 represent the first, second, third, and fourth quartiles of hormone levels, respectively

To enhance clinical interpretability, we categorized sex hormone levels by quartiles and analyzed "aging acceleration" (BAA > 0) as the outcome. In female participants, higher estradiol levels were correlated with aging deceleration, indicating an anti-aging effect. Compared with the first quartile (Q1), the odds ratios (OR) for Q2, Q3, and Q4 were 0.82 (95% CI: 0.73—0.94), 0.77 (95% CI: 0.68—0.88), and 0.62 (95% CI: 0.54—0.71), respectively (eTable 2, Fig. 1). In contrast, increased testosterone levels were correlated with aging acceleration. Participants in the highest quartile (Q4) of testosterone had a 21% higher risk of aging acceleration compared with those in the lowest quartile (Q1) (OR: 1.21, 95% CI: 1.06—1.39). (eTable 2, Fig. 1).

In male participants, higher testosterone levels were consistently associated with aging deceleration. Compared to Q1, the ORs for Q2, Q3, and Q4 were 0.79 (95% CI: 0.70—0.90), 0.77 (95% CI: 0.68—0.88), and 0.75 (95% CI: 0.65—0.85), respectively (eTable 2, Fig. 1).

### Age-stratified subgroup analysis

Following the analysis of the overall population, subgroup analyses were performed based on different age groups (eTable 3, eTable 4, eFigure 5, eFigure 6 in the supplement). The findings were consistent with those of the overall population: estradiol exerted an anti-aging effect in female participants, with the most significant impact observed in the 41–55 age group. In contrast, testosterone appeared to promote aging. In male participants, testosterone acted as an anti-aging factor, whereas estradiol did not exhibit a significant effect.

### Impact of HRT on BAA in females

**Fig. 2:**
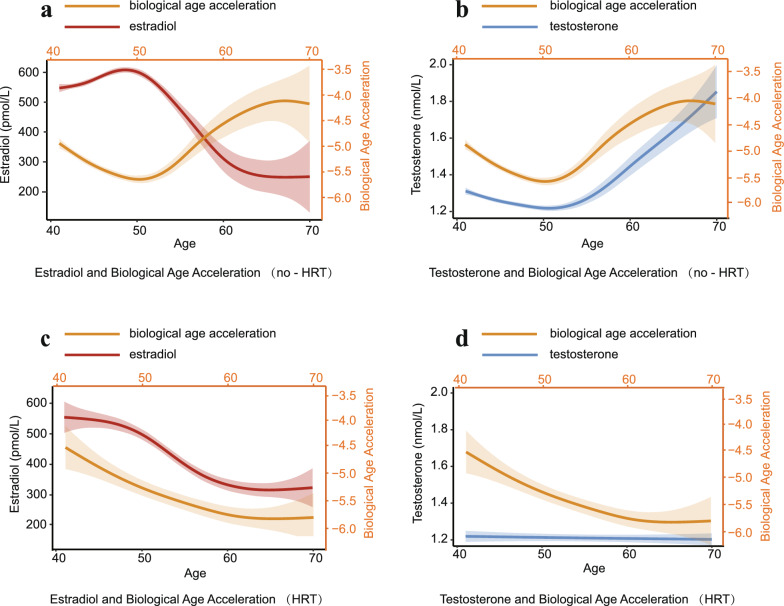
Female-specific trends in sex hormones and biological age acceleration: stratification by HRT status (**a**) estradiol and biological age acceleration (no-HRT) (**b**) testosterone and biological age acceleration (no-HRT) (**c**) estradiol and biological age acceleration (HRT) (**d**) testosterone and biological age acceleration (HRT) Abbreviations: HRT, hormone replacement therapy

The characteristics of the HRT and no-HRT groups are presented in eTable 5. In the no-hormone replacement therapy (HRT) group aged 41–50 years, estradiol levels stabilized at 500–600 pmol/L with a modest upward trend. After 50 years of age, estradiol levels declined markedly, reaching < 300 pmol/L by 60 years of age, after which they stabilized. During this period, aging deceleration weakened progressively. Notably, inflection points in the trajectories of BAA corresponded with changes in estradiol levels (Fig. 2 (a)). Testosterone levels gradually declined between 41 and 50 years of age, reaching a nadir at 50–55 years of age before rebounding (Fig. 2 (b)).

In the HRT group, estradiol levels mirrored the trends observed in the no-HRT group (a marked decline from 50–60 years of age, with stabilization post-60 years). However, aging deceleration in the HRT group increased steadily between 41 and 70 years of age, with no distinct inflection points, and was more pronounced than in the no-HRT group (HRT group BAA: -4.5 to -6.0; no-HRT group BAA: -4.0 to -5.5). (Fig. 2 (c)) Testosterone levels remain relatively stable between 41 and 70 years of age, with no upward trend observed (Fig. 2 (d)).

**Fig. 3:**
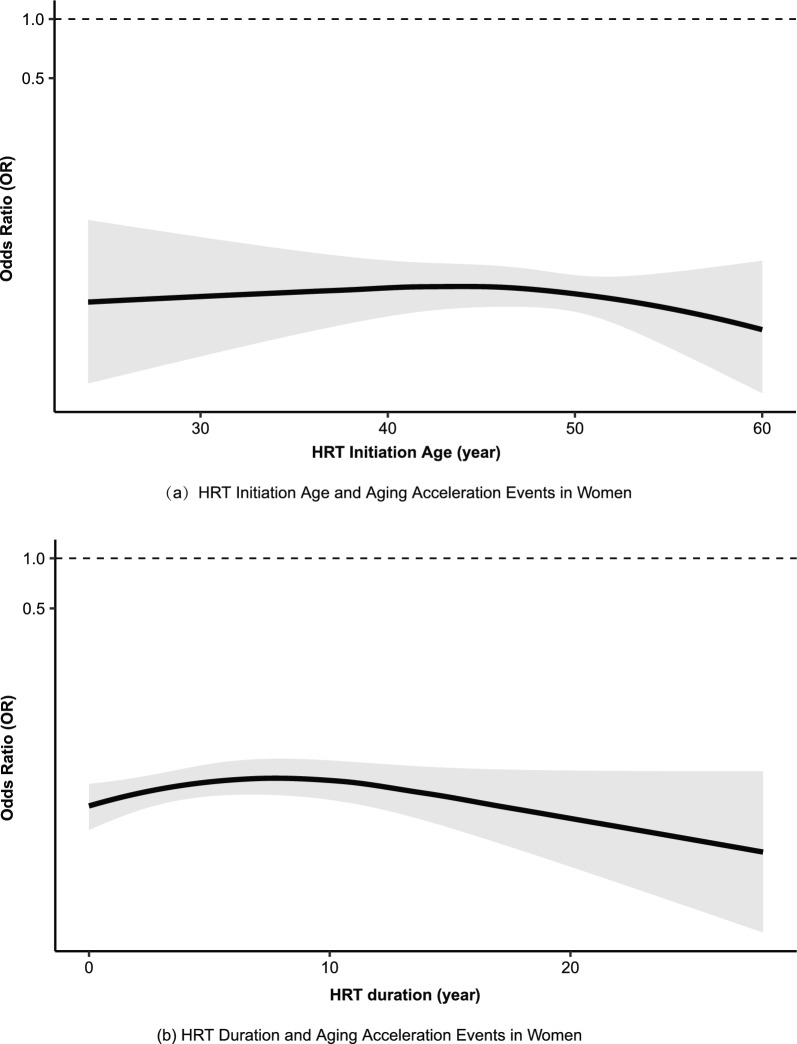
Association of HRT initiation age and duration with Aging Acceleration (a) HRT initiation age; (b) HRT duration. Estimates were adjusted for age, BMI, smoking status, drinking status, HRT initiation age (b), HRT duration (a). Solid lines are adjusted ORs, with the area showing 95% CIs derived from restricted cubic spline regressions with three knots. Abbreviations: HRT, hormone replacement therapy; OR, odd ratio

A total of 4,728 women with clearly defined HRT initiation and cessation times were included for further analysis. The average age of starting HRT was 48 years and the average treatment duration was 5 years. (eTable 6) In females receiving HRT, a significant deceleration of deceleration of aging was observed across the cohort, regardless of the age at treatment initiation (Fig. 3(a)).

When stratified by initiation age, the magnitude of aging deceleration varied, with the most pronounced effect observed among women who initiated HRT between 56 and 60 years of age. (eTable 7) In addition, a clear duration–response relationship was identified, whereby longer durations of HRT use were associated with greater degrees of aging deceleration (Fig. 3(b)).

## Discussion

This study quantified the impact of sex hormone reduction on aging across different age groups. Among middle-aged and elderly female participants, the 51—55 years of age group emerged as the most significant window for fluctuations in estradiol levels. During this period, per SD decrease in estradiol was associated with a BAA of 0.29 years in female participants. Conversely, in male participants, changes in testosterone levels after 56 years had the most pronounced impact on aging, especially in 66–70 years of age group. Results from the sensitivity analysis were consistent with this conclusion. (eTable 8) These findings suggest that sex hormone decline does not exert a uniform effect across the lifespan.

Our study reveals an interesting phenomenon, in females undergoing HRT, the relationship between testosterone and BAA shifted and the correlation between estradiol and BAA was weaker than in those not receiving estradiol supplementation. This attenuation may be attributed to more stable estradiol levels in the HRT group, compared to greater fluctuations in the untreated group.These findings indicate that sex hormones not only serve as aging biomarkers but may also regulate the aging process.

In this study, HRT initiated at age 47 resulted in lower baseline estradiol levels in females aged 41–50 compared to non-HRT counterparts, and the decline in estradiol was attenuated in females aged 50 and older. In the no-HRT group, testosterone levels rebounded after 50 years of age, while HRT users exhibited a persistent decline (Fig. 3). This divergence may arise from several factors: [1] estrogen depletion triggering gonadotropin-driven androgen overproduction, which is suppressed by HRT; [24, 25] [2] Estrogen deficiency exacerbates insulin resistance [26, 27] and triggering steroidogenesis. [28] Thus, the testosterone-suppressing effects of HRT may provide dual benefits: delaying aging and improving metabolic health.

Menopause-related estradiol decline increases the risk of diabetes, osteoporosis, and other disorders. [14, 15] HRT may counteract these effects through various mechanisms, such as reducing visceral adiposity, [29] improving insulin sensitivity, [26, 27] enhancing sleep quality, and promoting bone health. [30] The “critical period hypothesis” suggests that early HRT optimizes neuroprotective effects, [31] improves cognition [32, 33] and may reduce Alzheimer’s risk. [34] Females who start HRT early (< 50 years) appear to experience increased longevity, [35] reduced stroke risk. [36] However, the cardiovascular effects of HRT remain contentious. [37–39].

HRT offers clear benefits in alleviating menopausal symptoms, increasing bone density, reducing the risks of fractures and colorectal cancer, and lowering all-cause mortality. Our study also suggested that a longer duration of HRT is associated with greater benefits in reducing biological age. However, prolonged use of HRT may also elevate the risks of stroke, pulmonary embolism, and breast cancer. [40, 41] Therefore, HRT requires a careful, individualized risk–benefit assessment and ongoing hormonal monitoring.

Our study suggests the optimal timing for hormone therapy in females is around 50–55 years. However, similar recommendations cannot be made for men at this stage. Unlike estrogen therapy in females, which has well-documented effects on aging, testosterone therapy in men lacks consistent data to identify an optimal treatment window. What’s more, the potential benefits and risks of testosterone therapy in men are not yet fully defined. Currently, testosterone supplementation is primarily used to treat severe conditions such as cachexia, chronic kidney disease, [42] acquired immunodeficiency syndrome (AIDS), [43, 44] sepsis, [45] and burns. [46–49] Well-designed clinical trials are essential to establish safe guidelines for testosterone therapy in men.

For the calculation of biological age and BAA, we adopted the KDM Age. Though it is a robust predictor of morbidity and mortality, it is important to note that it may not fully capture aspects related to longevity. Biomarkers optimized for predicting mortality risk (such as those capturing inflammation or metabolic stress) differ from those associated with long-term genomic stability and healthy aging. Consequently, our findings that HRT decelerating biological aging should be interpreted as an effect on reducing the risk of adverse outcomes and improving health span, rather than a direct measure of extended lifespan.

In this analysis, several methodological factors should be considered when interpreting our findings. Estradiol levels in men and testosterone levels in females are often near the lower detection limit, which may introduce variability and reduce measurement precision. This variability leads to non-differential measurement error, typically biasing regression coefficients toward the null and decreasing statistical power. [50] As a result, the true associations between sex hormones and BAA could be stronger than observed. This attenuation bias is particularly important when interpreting null findings in certain sex-specific subgroups, such as the lack of significant associations between estradiol and biological age in men, which may reflect limited statistical power due to assay sensitivity constraints at low concentrations.

As an observational study, this research cannot distinguish between the direct anti-aging effects of HRT (e.g., estrogen receptor signaling) and indirect effects (e.g., vascular improvement or anti-inflammatory actions). In recent years, there has been growing research on the relationship between sex hormones and aging-related genes. Future studies integrating multi-omics data and experimental validation are needed to clarify key molecular pathways involved. Additionally, the participants in this study were drawn from a single database, which may not fully reflect the broader population. Further validation in diverse populations is required. Moreover, the lack of testosterone supplementation data in the database precludes an evaluation of the effectiveness of testosterone replacement therapy. In terms of statistical power, although our study was adequately powered for the primary analyses, further stratification by sex hormones resulted in smaller subgroups. Consequently, statistical power in these subgroups, especially in females aged 61–65 and 66–70 years, was reduced.

In conclusion, this study provides evidence for the significant temporal synchronization between changes in sex hormones (estradiol and testosterone) and BAA, highlighting their crucial yet distinct roles in the aging process. Notably, the findings quantify the impact of sex hormone reduction on aging across different ages, identifying the period most sensitive to sex hormone reduction and highlighting the optimal window for females to initiate HRT. The results support the hypothesis of critical periods for HRT. Overall, the results of this study provide a foundation for future research exploring the complex mechanisms through which sex hormones regulate the aging process.

## Supplementary Information

Additional file 1.

## Data Availability

UK Biobank data are available to all researchers for health-related research and public interest through registration on the UK Biobank (www.ukbiobank.ac.uk). This research has been conducted using the UK Biobank resource under application number 89483.

## References

[1] Kirkwood TB. Understanding the odd science of aging. Cell. 2005;120(4):437–47. 15734677 10.1016/j.cell.2005.01.027

[2] Kwon D, Belsky DW. A toolkit for quantification of biological age from blood chemistry and organ function test data: BioAge. GeroSci. 2021;43(6):2795–808. 10.1007/s11357-021-00480-5 PMC8602613 34725754

[3] Levine ME. Modeling the rate of senescence: can estimated biological age predict mortality more accurately than chronological age? J Gerontol A Biol Sci Med Sci. 2013;68(6):667–74. 23213031 10.1093/gerona/gls233 PMC3660119

[4] Chen H, Yin J, Xiang Y, Zhang N, Huang Z, Zhang Y, et al. Alcohol consumption and accelerated biological ageing in middle-aged and older people: a longitudinal study from two cohorts. Addiction. 2024;119(8):1387–99. 38679855 10.1111/add.16501

[5] Galkin F, Kochetov K, Koldasbayeva D, Faria M, Fung HH, Chen AX, et al. Psychological factors substantially contribute to biological aging: evidence from the aging rate in Chinese older adults. Aging. 2022;14(18):7206–22. 36170009 10.18632/aging.204264 PMC9550255

[6] Dmitrieva NI, Gagarin A, Liu D, Wu CO, Boehm M. Middle-age high normal serum sodium as a risk factor for accelerated biological aging, chronic diseases, and premature mortality. EBioMed. 2023;87:104404. 10.1016/j.ebiom.2022.104404 PMC9873684 36599719

[7] You Y, Chen Y, Liu R, Zhang Y, Wang M, Yang Z, et al. Inverted U-shaped relationship between sleep duration and phenotypic age in US adults: a population-based study. Sci Rep. 2024;14(1):6247. 38486063 10.1038/s41598-024-56316-7 PMC10940593

[8] Debès C, Papadakis A, Grönke S, Karalay Ö, Tain LS, Mizi A, et al. Ageing-associated changes in transcriptional elongation influence longevity. Nat. 2023;616(7958):814–21. 10.1038/s41586-023-05922-y PMC10132977 37046086

[9] Fox FAU, Liu D, Breteler MMB, Aziz NA. Physical activity is associated with slower epigenetic ageing-findings from the Rhineland study. Aging Cell. 2023;22(6):e13828. 37036021 10.1111/acel.13828 PMC10265180

[10] Randolph JF Jr., Sowers M, Bondarenko IV, Harlow SD, Luborsky JL, Little RJ. Change in estradiol and follicle-stimulating hormone across the early menopausal transition: effects of ethnicity and age. J Clin Endocrinol Metab. 2004;89(4):1555–61. 15070912 10.1210/jc.2003-031183

[11] Pataky MW, Young WF, Nair KS. Hormonal and metabolic changes of aging and the influence of lifestyle modifications. Mayo Clin Proc. 2021;96(3):788–814. 33673927 10.1016/j.mayocp.2020.07.033 PMC8020896

[12] Liu Y, Li C. Hormone therapy and biological aging in postmenopausal women. JAMA Netw Open. 2024;7(8):e2430839. 39207753 10.1001/jamanetworkopen.2024.30839 PMC11362863

[13] Kusters CDJ, Paul KC, Lu AT, Ferruci L, Ritz BR, Binder AM, et al. Higher testosterone and testosterone/estradiol ratio in men are associated with decreased Pheno-/GrimAge and DNA-methylation based PAI1. GeroSci. 2024;46(1):1053–69. 10.1007/s11357-023-00832-3 PMC10828310 37369886

[14] Stepan JJ, Hruskova H, Kverka M. Update on menopausal hormone therapy for fracture prevention. Curr Osteoporos Rep. 2019;17(6):465–73. 31741221 10.1007/s11914-019-00549-3 PMC6944675

[15] Ren Y, Zhang M, Liu Y, Sun X, Wang B, Zhao Y, et al. Association of menopause and type 2 diabetes mellitus. Menopause (New York, NY). 2019;26(3):325–30. 10.1097/GME.0000000000001200 30130291

[16] Wilkinson HN, Hardman MJ. A role for estrogen in skin ageing and dermal biomechanics. Mech Ageing Dev. 2021;197:111513. 34044023 10.1016/j.mad.2021.111513

[17] Yeap BB, Marriott RJ, Dwivedi G, Adams RJ, Antonio L, Ballantyne CM, et al. Associations of testosterone and related hormones with all-cause and cardiovascular mortality and incident cardiovascular disease in men: individual participant data meta-analyses. Ann Intern Med. 2024;177(6):768–81. 38739921 10.7326/M23-2781 PMC12768424

[18] Lehallier B, Gate D, Schaum N, Nanasi T, Lee SE, Yousef H, et al. Undulating changes in human plasma proteome profiles across the lifespan. Nat Med. 2019;25(12):1843–50. 31806903 10.1038/s41591-019-0673-2 PMC7062043

[19] Shen X, Wang C, Zhou X, Zhou W, Hornburg D, Wu S, et al. Nonlinear dynamics of multi-omics profiles during human aging. Nat Aging. 2024;4(11):1619–34. 39143318 10.1038/s43587-024-00692-2 PMC11564093

[20] Sudlow C, Gallacher J, Allen N, Beral V, Burton P, Danesh J, et al. UK Biobank: an open access resource for identifying the causes of a wide range of complex diseases of middle and old age. PLoS Med. 2015;12(3):e1001779. 25826379 10.1371/journal.pmed.1001779 PMC4380465

[21] Klemera P, Doubal S. A new approach to the concept and computation of biological age. Mech Ageing Dev. 2006;127(3):240–8. 16318865 10.1016/j.mad.2005.10.004

[22] Gao X, Geng T, Jiang M, Huang N, Zheng Y, Belsky DW, et al. Author Correction: Accelerated biological aging and risk of depression and anxiety: evidence from 424,299 UK Biobank participants. Nat Commun. 2023;14(1):5970. 37749135 10.1038/s41467-023-41786-6 PMC10519949

[23] Hao M, Zhang H, Wu J, Huang Y, Li X, Wang M, et al. Gompertz Law-Based Biological Age (GOLD BioAge): A Simple and Practical Measurement of Biological Ageing to Capture Morbidity and Mortality Risks. Adv Sci (Weinh). 2025;12(32):e01765. 40600465 10.1002/advs.202501765 PMC12407260

[24] Judd HL, Lucas WE, Yen SS. Effect of oophorectomy on circulating testosterone and androstenedione levels in patients with endometrial cancer. Am J Obstet Gynecol. 1974;118(6):793–8. 4815860 10.1016/0002-9378(74)90490-6

[25] Davison SL, Bell R, Donath S, Montalto JG, Davis SR. Androgen levels in adult females: changes with age, menopause, and oophorectomy. J Clin Endocrinol Metab. 2005;90(7):3847–53. 15827095 10.1210/jc.2005-0212

[26] Salpeter SR, Walsh JM, Ormiston TM, Greyber E, Buckley NS, Salpeter EE. Meta-analysis: effect of hormone-replacement therapy on components of the metabolic syndrome in postmenopausal women. Diabetes Obes Metab. 2006;8(5):538–54. 16918589 10.1111/j.1463-1326.2005.00545.x

[27] Margolis KL, Bonds DE, Rodabough RJ, Tinker L, Phillips LS, Allen C, et al. Effect of oestrogen plus progestin on the incidence of diabetes in postmenopausal women: results from the women’s health initiative hormone trial. Diabetologia. 2004;47(7):1175–87. 15252707 10.1007/s00125-004-1448-x

[28] Dunaif A. Insulin resistance and the polycystic ovary syndrome: mechanism and implications for pathogenesis. Endocr Rev. 1997;18(6):774–800. 9408743 10.1210/edrv.18.6.0318

[29] Papadakis GE, Hans D, Gonzalez Rodriguez E, Vollenweider P, Waeber G, Marques-Vidal P, et al. Menopausal hormone therapy is associated with reduced total and visceral adiposity: the OsteoLaus cohort. J Clin Endocrinol Metab. 2018;103(5):1948–57. 29596606 10.1210/jc.2017-02449

[30] Liu T, Li N, Yan YQ, Liu Y, Xiong K, Liu Y, et al. Recent advances in the anti-aging effects of phytoestrogens on collagen, water content, and oxidative stress. Phytotherapy res PTR. 2020;34(3):435–47. 10.1002/ptr.6538 PMC7078862 31747092

[31] Ma Y, Liu M, Yang L, Zhang L, Guo H, Qin P, et al. Loss of estrogen efficacy against Hippocampus damage in long-term OVX mice is related to the reduction of Hippocampus local estrogen production and estrogen receptor degradation. Mol Neurobiol. 2020;57(8):3540–51. 32542593 10.1007/s12035-020-01960-z

[32] Maki PM. Hormone therapy and cognitive function: is there a critical period for benefit? Neurosci. 2006;138(3):1027–30. 10.1016/j.neuroscience.2006.01.001 16488547

[33] MacLennan AH, Henderson VW, Paine BJ, Mathias J, Ramsay EN, Ryan P, et al. Hormone therapy, timing of initiation, and cognition in women aged older than 60 years: the remember pilot study. Menopause. 2006;13(1):28–36. 16607096 10.1097/01.gme.0000191204.38664.61

[34] Resnick SM, Henderson VW. Hormone therapy and risk of Alzheimer disease: a critical time. JAMA. 2002;288(17):2170–2. 12413378 10.1001/jama.288.17.2170

[35] Brandts L, van Poppel FWA, van den Brandt PA. Female reproductive factors and the likelihood of reaching the age of 90 years. The Netherlands Cohort Study. Maturitas. 2019;125:70–80. 31133221 10.1016/j.maturitas.2019.04.213

[36] Carrasquilla GD, Frumento P, Berglund A, Borgfeldt C, Bottai M, Chiavenna C, et al. Postmenopausal hormone therapy and risk of stroke: a pooled analysis of data from population-based cohort studies. PLoS Med. 2017;14(11):e1002445. 29149179 10.1371/journal.pmed.1002445 PMC5693286

[37] Boardman HM, Hartley L, Eisinga A, Main C, Roqué i Figuls M, Bonfill Cosp X, et al. Hormone therapy for preventing cardiovascular disease in post-menopausal women. Cochrane Database Syst Rev. 2015;2015(3):Cd002229. 25754617 10.1002/14651858.CD002229.pub4 PMC10183715

[38] Rosano GM, Vitale C, Fini M. Hormone replacement therapy and cardioprotection: what is good and what is bad for the cardiovascular system? Ann N Y Acad Sci. 2006;1092:341–8. 17308159 10.1196/annals.1365.031

[39] Oliver-Williams C, Glisic M, Shahzad S, Brown E, Pellegrino Baena C, Chadni M, et al. The route of administration, timing, duration and dose of postmenopausal hormone therapy and cardiovascular outcomes in women: a systematic review. Hum Reprod Update. 2019;25(2):257–71. 30508190 10.1093/humupd/dmy039

[40] Rossouw JE, Anderson GL, Prentice RL, LaCroix AZ, Kooperberg C, Stefanick ML, et al. Risks and benefits of estrogen plus progestin in healthy postmenopausal women: principal results from the Women’s Health Initiative randomized controlled trial. JAMA. 2002;288(3):321–33. 12117397 10.1001/jama.288.3.321

[41] Genazzani AR, Monteleone P, Giannini A, Simoncini T. Hormone therapy in the postmenopausal years: considering benefits and risks in clinical practice. Hum Reprod Update. 2021;27(6):1115–50. 34432008 10.1093/humupd/dmab026

[42] Johansen KL, Mulligan K, Schambelan M. Anabolic effects of nandrolone decanoate in patients receiving dialysis: a randomized controlled trial. JAMA. 1999;281(14):1275–81. 10208142 10.1001/jama.281.14.1275

[43] Johns K, Beddall MJ, Corrin RC. Anabolic steroids for the treatment of weight loss in HIV-infected individuals. Cochrane Database Syst Rev. 2005;4:Cd005483. 10.1002/14651858.CD005483 PMC12174972 16235407

[44] Ferrando SJ, Freyberg Z. Treatment of depression in HIV positive individuals: a critical review. Int rev psych (Abingdon, England). 2008;20(1):61–71. 10.1080/09540260701862060 18240063

[45] Fourrier F, Jallot A, Leclerc L, Jourdain M, Racadot A, Chagnon JL, et al. Sex steroid hormones in circulatory shock, sepsis syndrome, and septic shock. Circ Shock. 1994;43(4):171–8. 7895322

[46] Semple CG, Mitchell R, Hollis S, Robertson WR. An investigation of LH pulsatility in burned men by bioassay and radioimmunoassay. Acta Endocrinol Copenh. 1992;126(5):404–9. 1621483 10.1530/acta.0.1260404

[47] Plymate SR, Vaughan GM, Mason AD, Pruitt BA. Central hypogonadism in burned men. Horm Res. 1987;27(3):152–8. 3692444 10.1159/000180803

[48] Hart DW, Wolf SE, Ramzy PI, Chinkes DL, Beauford RB, Ferrando AA, et al. Anabolic effects of oxandrolone after severe burn. Ann Surg. 2001;233(4):556–64. 11303139 10.1097/00000658-200104000-00012 PMC1421286

[49] Knuth CM, Auger C, Jeschke MG. Burn-induced hypermetabolism and skeletal muscle dysfunction. Am J Physiol Cell Physiol. 2021;321(1):C58-c71. 33909503 10.1152/ajpcell.00106.2021 PMC8321793

[50] Gaye A, Peakman T, Tobin MD, Burton PR. Understanding the impact of pre-analytic variation in haematological and clinical chemistry analytes on the power of association studies. Int J Epidemiol. 2014;43(5):1633–44. 25085103 10.1093/ije/dyu127 PMC4190517

