# Supplementary material for: Divergent impacts of estradiol/testosterone reduction on biological aging: optimal HRT window in females recommended
Source: Biol Sex Differ. 2026 Mar 13;17:81. doi: 10.1186/s13293-026-00873-1 (PMC13097923; doi:10.1186/s13293-026-00873-1)
Supplement: Supplementary file 1 — Additional file 1. [file 13293_2026_873_MOESM1_ESM.docx]

**Supplemental Online Content**

**eTable 1.** Variables Used in the UK Biobank

**eTable 2.** Association between Sex Hormones and Aging Acceleration Events in Total Population

**eTable 3.** Association between Estradiol and Aging Acceleration Events by Sex and Age Group

**eTable 4.** Association between Testosterone and Aging Acceleration Events by Sex and Age Group

**eTable 5.** Baseline Characteristics of Females by HRT

**eTable 6.** Population Characteristics of HRT participants

**eTable 7.** Association between HRT Initiation Age and Biological Age Acceleration

**eTable 8.** Sensitivity Analysis Using the Gompertz law-based biological age model

**eFigure 1.** Flow Diagram of the Analytic Sample

**eFigure 2.** Age Distribution of Total Population

**eFigure 3.** Trends in Sex Hormones and Biological Age Acceleration in females

**eFigure 4.** Trends in Sex Hormones and Biological Age Acceleration in Males

**eFigure 5.** Association between Sex Hormones and Aging Acceleration Events in Females by Age Group

**eFigure 6.** Association between Sex Hormones and Aging Acceleration Events in Males by Age Group

**eTable1. Definitions of Variables Used in the UK Biobank**

| **Variable** | **Units** | [**Value Type**](https://biobank.ndph.ox.ac.uk/ukb/help.cgi?cd=value_type) | **Definition in this study** | **UK Biobank Data-Field ID** |
| --- | --- | --- | --- | --- |
| **Sex** | -- | Categorical | 1=Male 0=Female | 31 |
| **Smoking status** | -- | Categorical | 0=Never 1=Previous 2=Current -3=Prefer not to answer | 20116 |
| **Drinking status** | -- | Categorical | 0=Never 1=Previous 2=Current -3=Prefer not to answer | 20117 |
| **Ever used HRT** | -- | Categorical | 1=Yes 0=No | 2814 |
| **age** | years | Integer | -- | 21022 |
| **Systolic blood pressure** | mmHg | Integer | -- | 4080 |
| **Age started HRT** | years | Integer, years | -- | 3536 |
| **Age last used HRT** |  | Integer, years | -- | 3546 |
| **Date of attending assessment centre** | -- | Date | -- | 53 |
| **BMI** | kg/m2 | Continuous | -- | 21001 |
| **Waist circumference** | cm | Continuous | -- | 48 |
| **Hip circumference** | cm | Continuous | -- | 49 |
| [**Glucose**](https://biobank.ndph.ox.ac.uk/ukb/field.cgi?id=30740) | mmol/L | Continuous | -- | 30740 |
| **Glycated haemoglobin** | mmol/L | Continuous | -- | 30750 |
| **HDL** | mmol/L | Continuous | -- | 30760 |
| **LDL** | mmol/L | Continuous | -- | 30780 |
| [**Triglycerides**](https://biobank.ndph.ox.ac.uk/ukb/field.cgi?id=30870) | mmol/L | Continuous | -- | 30870 |
| [**Cholesterol**](https://biobank.ndph.ox.ac.uk/ukb/field.cgi?id=30690) | mmol/L | Continuous | -- | 30690 |
| **FEV1** | L | Continuous | -- | 3063 |
| [**Albumin**](https://biobank.ndph.ox.ac.uk/ukb/field.cgi?id=30600) | g/L | Continuous | -- | 30600 |
| [**Alkaline phosphatase**](https://biobank.ndph.ox.ac.uk/ukb/field.cgi?id=30610) | U/L | Continuous | -- | 30610 |
| [**Urea**](https://biobank.ndph.ox.ac.uk/ukb/field.cgi?id=30670) | mmol/L | Continuous | -- | 30670 |
| [**Creatinine**](https://biobank.ndph.ox.ac.uk/ukb/field.cgi?id=30700) | umol/L | Continuous | -- | 30700 |
| [**Urate**](https://biobank.ndph.ox.ac.uk/ukb/field.cgi?id=30880) | umol/L | Continuous | -- | 30880 |
| [**C-reactive protein**](https://biobank.ndph.ox.ac.uk/ukb/field.cgi?id=30710) | mg/L | Continuous | -- | 30710 |
| [**White blood cell (leukocyte) count**](https://biobank.ndph.ox.ac.uk/ukb/field.cgi?id=30000) | 10^9^/L | Continuous | -- | 30000 |
| [**Lymphocyte count**](https://biobank.ndph.ox.ac.uk/ukb/field.cgi?id=30120) | 10^9^/L | Continuous | -- | 30120 |
| [**Lymphocyte percentage**](https://biobank.ndph.ox.ac.uk/ukb/field.cgi?id=30180) | % | Continuous | -- | 30180 |
| [**Red blood cell (erythrocyte) count**](https://biobank.ndph.ox.ac.uk/ukb/field.cgi?id=30010) | 10^12^/L | Continuous | -- | 30010 |
| **Mean sphered cell volume** | fl | Continuous | -- | 30270 |
| **Red blood cell (erythrocyte) distribution width** | % | Continuous | -- | 30070 |
| **SHBG** | nmol/L | Continuous | -- | 30830 |
| **testosterone** | nmol/L | Continuous | -- | 30850 |
| **estradiol** | pmol/L | Continuous | -- | 30800 |

Abbreviations: HRT, hormone-replacement therapy；BMI，Body Mass Index；HDL，high-density lipoprotein；LDL, low-density lipoprotein；FEV1, Forced Expiratory Volume in 1 second.

**eTable2. Association between Sex hormones and** **Aging Acceleration Events in Total Population**

| **Gender** |  | **Estradiol, pmol/L** | | | | **Per-SD decrease of lnest** |
| --- | --- | --- | --- | --- | --- | --- |
|  |  | **Q1** | **Q2** | **Q3** | **Q4** |  |
| **Female** | **No.of age acceleration** | 618/9827 | 465/9826 | 429/9825 | 321/9825 | -- |
|  | **Unadjusted** | -- | 0.74 (0.65 to 0.84) | 0.68 (0.60 to 0.77) | 0.50 (0.44 to 0.58) | 1.23 (1.25-1.39) |
|  | **Model1 ^a^** | -- | 0.82 (0.73 to 0.94) | 0.77 (0.68 to 0.88) | 0.62 (0.54 to 0.71) | 1.15 (1.15-1.28) |
| **Male** | **No.of age acceleration** | 543/3864 | 557/3849 | 574/3828 | 628/3842 | -- |
|  | **Unadjusted** | -- | 1.03 (0.91 to 1.18) | 1.08 (0.95 to 1.23) | 1.20 (1.05 to 1.35) | 0.92 (0.91-0.99) |
|  | **Model1** | -- | 1.04 (0.91 to 1.18) | 1.04 (0.91 to 1.18) | 1.14 (1.00 to 1.30) | 0.94 (0.92-1.01) |
|  |  | **Testosterone, nmol/L** | | | | **Per-SD decrease of lntes** |
|  |  | **Q1** | **Q2** | **Q3** | **Q4** |  |
| **Female** | **No.of age acceleration** | 374/9838 | 428/9836 | 440/9804 | 591/9825 | -- |
|  | **Unadjusted** | -- | 1.15 (1.00 to 1.33) | 1.19 (1.03 to 1.37) | 1.62 (1.42 to 1.85) | 0.83 (0.80-0.87) |
|  | **Model1** | -- | 1.08 (0.94 to 1.25) | 1.04 (0.90 to 1.20) | 1.21 (1.06 to 1.39) | 0.93 (0.89-0.98) |
| **Male** | **No.of age acceleration** | 800/3846 | 556/3847 | 499/3845 | 447/3845 | -- |
|  | **Unadjusted** | -- | 0.64 (0.57 to 0.72) | 0.57 (0.50 to 0.64) | 0.50 (0.44 to 0.57) | 1.28 (1.23-1.34) |
|  | **Model1** | -- | 0.79 (0.70 to 0.90) | 0.77 (0.68 to 0.88) | 0.75 (0.65 to 0.85) | 1.10 (1.05-1.15) |

Abbreviations: CI: Confidence interval; OR: Odds ratio; SD: Standard deviation; lntes, log(testosterone); lnest, log(estradiol); Aging Acceleration, biological age acceleration ≥ 0.

Note:

^a^ generalized linear Model 1 included covariates of Smoking Status, Drinking Status and BMI.

Estradiol (pmol/L): Q1-Q4 in female respectively represent the ranges (175. 00 - 271.19), (271.20 - 407.69), (407.70 - 653.49), and (653.50 -8910.50). Q1-Q4 in male respectively represent the ranges (175. 00 - 188.79), (188.80 - 204.09), (204.10 - 230.89), and (230.90 -1775.20).

Testosterone (nmol/L): Q1-Q4 in female respectively represent the ranges (0.35 - 0.83), (0.84 - 1.14), (1.15 - 1.51), and (1.52 - 31.42). Q1-Q4 in male respectively represent the ranges (0.39 - 10.50)；(10.51 - 12.88)；(12.89 - 15.68)；(15.69 - 53.14).

**eTable 3. Association between Estradiol and Aging Acceleration Events by Sex and Age Group**

| **Gender** | **Age Group, y** |  | **Estradiol Levels, pmol/L** | | | | **Per-SD decrease of lnest** |
| --- | --- | --- | --- | --- | --- | --- | --- |
|  |  |  | **Q1** | **Q2** | **Q3** | **Q4** |  |
| Female | 41-45 | No.of age acceleration | 204/3374 | 183/4060 | 207/4241 | 142/4062 | -- |
|  |  | Model1 ^a^ | -- | 0.81 (0.65-1.00) | 0.90 (0.73-1.11) | 0.70 (0.55-0.87) | 1.15 (1.06-1.25) |
|  | 46-50 | No.of age acceleration | 152/2912 | 163/3415 | 142/3656 | 116/4066 | -- |
|  |  | Model1 | -- | 0.95 (0.75-1.20) | 0.78 (0.62-1.00) | 0.63 (0.48-0.81) | 1.23 (1.12-1.35) |
|  | 51-55 | No.of age acceleration | 118/1836 | 91/1637 | 62/1487 | 54/1485 | -- |
|  |  | Model1 | -- | 0.91 (0.68-1.22) | 0.66 (0.47-0.90) | 0.60 (0.43-0.84) | 1.25 (1.10-1.41) |
|  | 56-60 | No.of age acceleration | 79/821 | 17/425 | 11/278 | 5/141 | -- |
|  |  | Model1 | -- | 0.44 (0.25-0.75) | 0.45 (0.22-0.83) | 0.41 (0.14-0.96) | 1.52 (1.18-1.96) |
|  | 61-65 | No.of age acceleration | 33/599 | 6/196 | 4/119 | 4/48 | -- |
|  |  | Model1 | -- | 0.62 (0.23-1.43) | 0.80 (0.23-2.12) | 1.96 (0.55-5.43) | 0.89 (0.67-1.18) |
|  | 66-70 | No.of age acceleration | 32/285 | 5/93 | 3/44 | 0/23 | -- |
|  |  | Model1 | -- | 0.52 (0.17-1.29) | 0.65 (0.15-2.00) | 0 (--) | 1.42 (0.92-2.19) |
| Male | 41-45 | No.of age acceleration | 66/437 | 71/428 | 52/395 | 94/515 | -- |
|  |  | Model1 | -- | 1.18 (0.81-1.72) | 0.88 (0.58-1.32) | 1.28 (0.90-1.84) | 0.93 (0.82-1.05) |
|  | 46-50 | No.of age acceleration | 80/526 | 87/504 | 85/496 | 80/607 | -- |
|  |  | Model1 | -- | 1.13 (0.80-1.59) | 1.07 (0.76-1.52) | 0.83 (0.59-1.17) | 1.17 (1.02-1.35) |
|  | 51-55 | No.of age acceleration | 67/571 | 77/590 | 112/607 | 104/614 | -- |
|  |  | Model1 | -- | 1.16 (0.81-1.66) | 1.62 (1.16-2.27) | 1.43 (1.02-2.01) | 0.89 (0.80-0.99) |
|  | 56-60 | No.of age acceleration | 104/755 | 89/670 | 104/750 | 102/682 | -- |
|  |  | Model1 | -- | 0.98 (0.72-1.33) | 0.92 (0.68-1.24) | 0.99 (0.73-1.34) | 1.01 (0.91-1.13) |

| **Gender** | **Age Group, y** |  | **Estradiol Levels, pmol/L** | | | | **Per-SD decrease of lnest** |
| --- | --- | --- | --- | --- | --- | --- | --- |
|  |  |  | **Q1** | **Q2** | **Q3** | **Q4** |  |
| Male | 61-65 | No.of age acceleration | 125/883 | 141/986 | 132/940 | 141/812 | -- |
|  |  | Model1 | -- | 1.01 (0.78-1.32) | 0.97 (0.74-1.27) | 1.24 (0.95-1.62) | 0.96 (0.88-1.05) |
|  | 66-70 | No.of age acceleration | 101/692 | 92/671 | 89/640 | 107/612 | -- |
|  |  | Model1 | -- | 0.91 (0.67-1.25) | 0.95 (0.69-1.30) | 1.17 (0.86-1.58) | 0.90 (0.82-1.00) |

Abbreviations: CI: Confidence interval; OR: Odds ratio; SD: Standard deviation; lntes, log(testosterone); lnest, log(estradiol); Aging Acceleration, biological age acceleration ≥ 0.

Notes:

^a^ generalized linear Model 1 included covariates of Smoking Status, Drinking Status and BMI.

Estradiol quartile cutoffs (pmol/L) for females: 41-45y (Q1: 175-288, Q2: 288-425, Q3: 425-666, Q4: 666-7686); 46-50y (Q1: 175-293, Q2: 293-443, Q3: 443-720, Q4: 720-8911); 51-55y (Q1: 175-256, Q2: 256-382, Q3: 382-624, Q4: 624-6085); 56-60y (Q1: 175-212, Q2: 212-273, Q3: 273-410, Q4: 410-4496); 61-65y (Q1: 175-201, Q2: 201-235, Q3: 235-336, Q4: 336-2499); 66-70y (Q1: 175-195, Q2: 195-230, Q3: 230-332, Q4: 332-2838).

Estradiol quartile cutoffs (pmol/L) for males: 41-45y (Q1: 175-189, Q2: 189-205, Q3: 205-237, Q4: 237-1484); 46-50y (Q1: 175-189, Q2: 189-205, Q3: 205-238, Q4: 238-1029); 51-55y (Q1: 175-189, Q2: 189-205, Q3: 205-232, Q4: 232-1775); 56-60y (Q1: 175-188, Q2: 188-204, Q3: 204-230, Q4: 230-1230); 61-65y (Q1: 175-189, Q2: 189-203, Q3: 203-227, Q4: 227-1626); 66-70y (Q1: 175-188, Q2: 188-203, Q3: 203-228, Q4: 228-1756).

**eTable 4. Association between Testosterone and** **Aging Acceleration Events by Sex and Age Group**

| **Gender** | **Age Group, y** |  | **Testosterone Levels (nmol/L)** | | | | **Per-SD decrease of lntes** |
| --- | --- | --- | --- | --- | --- | --- | --- |
|  |  |  | **Q1** | **Q2** | **Q3** | **Q4** |  |
| Female | 41-45 | No.of age acceleration | 123/3442 | 178/3831 | 160/4145 | 275/4319 | -- |
|  |  | Model1^a^ | -- | 1.25 (0.98-1.59) | 0.91 (0.71-1.16) | 1.37 (1.10-1.73) | 0.90 (0.83-0.97) |
|  | 46-50 | No.of age acceleration | 114/3502 | 137/3609 | 153/3564 | 169/3374 | -- |
|  |  | Model1 | -- | 1.10 (0.85-1.43) | 1.22 (0.94-1.57) | 1.21 (0.94-1.56) | 0.94 (0.87-1.03) |
|  | 51-55 | No.of age acceleration | 80/1777 | 72/1659 | 86/1567 | 87/1442 | -- |
|  |  | Model1 | -- | 0.90 (0.64-1.25) | 1.05 (0.76-1.45) | 1.03 (0.75-1.42) | 0.97 (0.86-1.09) |
|  | 56-60 | No.of age acceleration | 40/600 | 19/398 | 23/292 | 30/375 | -- |
|  |  | Model1 | -- | 0.7 (0.39-1.22) | 1.06 (0.61-1.82) | 0.83 (0.49-1.39) | 1.05 (0.86-1.29) |
|  | 61-65 | No.of age acceleration | 11/360 | 11/247 | 9/162 | 16/193 | -- |
|  |  | Model1 | -- | 1.37 (0.57-3.28) | 1.69 (0.66-4.23) | 1.94 (0.84-4.60) | 0.80 (0.60-1.07) |
|  | 66-70 | No.of age acceleration | 6/157 | 11/92 | 9/74 | 14/122 | -- |
|  |  | Model1 | -- | 3.03 (1.10-9.14) | 2.75 (0.91-8.77) | 2.41 (0.89-7.22) | 0.76 (0.55-1.05) |
| Male | 41-45 | No.of age acceleration | 90/375 | 62/423 | 66/472 | 65/505 | -- |
|  |  | Model1 | -- | 0.70 (0.48-1.02) | 0.78 (0.53-1.14) | 0.83 (0.57-1.23) | 1.05 (0.92-1.21） |
|  | 46-50 | No.of age acceleration | 117/523 | 85/522 | 70/521 | 60/567 | -- |
|  |  | Model1 | -- | 0.82 (0.59-1.13) | 0.75 (0.53-1.06) | 0.63 (0.44-0.91) | 1.19 (1.06-1.35) |
|  | 51-55 | No.of age acceleration | 122/597 | 78/596 | 80/611 | 80/578 | -- |
|  |  | Model1 | -- | 0.77 (0.56-1.06) | 0.79 (0.57-1.09) | 0.95 (0.68-1.33) | 1.01 (0.90-1.14) |
|  | 56-60 | No.of age acceleration | 136/731 | 102/731 | 92/702 | 69/693 | -- |
|  |  | Model1 | -- | 0.89 (0.66-1.19) | 0.94 (0.69-1.27) | 0.75 (0.53-1.04) | 1.09 (0.98-1.22) |

| **Gender** | **Age Group, y** |  | **Testosterone Levels, nmol/L** | | | | **Per-SD decrease of lntes** |
| --- | --- | --- | --- | --- | --- | --- | --- |
|  |  |  | **Q1** | **Q2** | **Q3** | **Q4** |  |
| Male | 61-65 | No.of age acceleration | 190/926 | 131/919 | 110/879 | 108/897 | -- |
|  |  | Model1 | -- | 0.76 (0.59-0.98) | 0.72 (0.55-0.94) | 0.73 (0.56-0.96) | 1.09 (0.99-1.20) |
|  | 66-70 | No.of age acceleration | 145/694 | 98/656 | 81/660 | 65/605 | -- |
|  |  | Model1 | -- | 0.80 (0.59-1.06) | 0.67 (0.49-0.91) | 0.63 (0.45-0.87) | 1.18 (1.05-1.31) |

Abbreviations: CI: Confidence interval; OR: Odds ratio; SD: Standard deviation; lntes, log(testosterone); lnest, log(estradiol); Aging Acceleration, biological age acceleration ≥ 0.

Notes:

^a^ generalized linear Model 1 included covariates of Smoking Status, Drinking Status and BMI.

Testosterone quartile cutoffs (nmol/L) for females: 41-45y (Q1: 0.35-0.89, Q2: 0.89-1.20, Q3: 1.20-1.57, Q4: 1.57-17.57); 46-50y (Q1: 0.35-0.84, Q2: 0.84-1.14, Q3: 1.14-1.51, Q4: 1.51-13.24);

51-55y (Q1: 0.35-0.81, Q2: 0.81-1.11, Q3: 1.11-1.46, Q4: 1.46-16.72); 56-60y (Q1: 0.35-0.71, Q2: 0.71-1.02, Q3: 1.02-1.45, Q4: 1.45-29.42); 61-65y (Q1: 0.36-0.68, Q2: 0.68-0.99, Q3: 0.99-1.41, Q4: 1.41-31.42); 66-70y (Q1: 0.35-0.71, Q2: 0.71-1.08, Q3: 1.08-1.58, Q4: 1.58-22.79).

Testosterone quartile cutoffs (nmol/L) for males: 41-45y (Q1:0.74-10.92, Q2:10.92-13.39, Q3:13.39-16.20, Q4:16.20-48.46); 46-50y (Q1: 0.81-10.55, Q2: 10.55-12.98, Q3: 12.98-15.89, Q4: 15.89-52.43); 51-55y (Q1: 0.50-10.51, Q2: 10.51-12.89, Q3: 12.89-15.57, Q4: 15.57-53.14); 56-60y (Q1: 0.89-10.42, Q2: 10.42-12.79, Q3: 12.79-15.54, Q4: 15.54-43.33); 61-65y (Q1: 0.39-10.46, Q2: 10.46-12.79, Q3: 12.79-15.67, Q4: 15.67-47.35); 66-70y (Q1: 0.95-10.37, Q2: 10.37-12.71, Q3: 12.71-15.43, Q4: 15.43-51.17).

**eTable5.** **Baseline Characteristics of Females by HRT**

|  | **nHRT** | **HRT** | **P value** |
| --- | --- | --- | --- |
| **n** | 34,272 | 5,031 | -- |
| **Age, y** | 46.00 [43.00, 49.00] | 54.00 [50.00, 60.00] | <0.001 |
| **Smoking, n (%)** | -- | -- | -- |
| **prefer not to answer** | 78 (0.2) | 16 (0.3) | <0.001 |
| **never** | 22086 (64.4) | 2676 (53.2) | -- |
| **previous** | 8652 (25.2) | 1824 (36.3) | -- |
| **current** | 3456 (10.1) | 515 (10.2) | -- |
| **Drinking, n (%)** | -- | -- | -- |
| **prefer not to answer** | 22 (0.1) | 8 (0.2) | <0.001 |
| **never** | 1557 (4.5) | 156 (3.1) | -- |
| **previous** | 924 (2.7) | 171 (3.4) | -- |
| **current** | 31769 (92.7) | 4696 (93.3) | -- |
| **SBP, mm Hg** | 123.50 [114.00, 135.00] | 131.50 [119.50, 144.50] | <0.001 |
| **BMI, kg/m2** | 25.41 [22.86, 29.14] | 25.97 [23.45, 29.83] | <0.001 |
| **Waist circumference, cm** | 80.00 [73.00, 89.00] | 82.00 [75.00, 92.00] | <0.001 |
| **Hip circumference, cm** | 101.00 [96.00, 108.00] | 102.00 [96.00, 109.00] | <0.001 |
| **Glucose, mmol/L** | 4.76 [4.47, 5.08] | 4.85 [4.53, 5.18] | <0.001 |
| **HbA1c, mmol/L** | 33.10 [30.90, 35.40] | 34.00 [31.80, 36.30] | <0.001 |
| **HDL-c, mmol/L** | 1.52 [1.30, 1.76] | 1.57 [1.34, 1.84] | <0.001 |
| **LDL-c, mmol/L** | 3.23 [2.78, 3.76] | 3.42 [2.90, 3.96] | <0.001 |
| **TG, mmol/L** | 1.06 [0.80, 1.50] | 1.30 [0.96, 1.82] | <0.001 |
| **Cholesterol, mmol/L** | 5.33 [4.74, 5.98] | 5.64 [5.01, 6.32] | <0.001 |
| **Testosterone, nmol/L** | 1.17 [0.86, 1.54] | 1.00 [0.71, 1.38] | <0.001 |
| **Estradiol, pmol/L** | 424.50 [281.00, 679.82] | 313.50 [226.90, 481.45] | <0.001 |
| **Age started hormone replacement therapy, y** | -- | 47.00 [42.00, 50.00] | -- |
| **Biological age, y** | 40.74 [37.69, 44.24] | 49.09 [44.26, 54.16] | <0.001 |
| **Biological age acceleration, y** | -5.47 [-7.41, -3.46] | -5.50 [-7.66, -3.36] | 0.087 |
| **Aging acceleration (%)** | 32710 (95.4) | 4760 (94.6) | 0.01 |

^a^ The description of continuous variables is represented by median and interquartile range, while the description of categorical variables is represented by n (%).

^b^ Group differences were compared using Student t test, χ2 test, or Wilcoxon rank test.

Abbreviations: HRT, hormone replacement therapy; SBP, Systolic blood pressure; BMI, Body Mass Index; HbA1c, glycated haemoglobin A1c；HDL-c, High density lipoprotein cholesterol; LDL-c, High density lipoprotein cholesterol; TG, Triglycerides.

**eTable6. Population Characteristics of HRT participants**

|  | **Overall** |
| --- | --- |
| **n** | 4,728 |
| **Age, y** | 54.00 [50.00, 59.00] |
| **Age_group, y** | -- |
| **41-45** | 361 (7.6) |
| **46-50** | 886 (18.7) |
| **51-55** | 1478 (31.3) |
| **56-60** | 1066 (22.5) |
| **61-65** | 680 (14.4) |
| **66-70** | 257 (5.4) |
| **SBP, mm Hg** | 131.00 [119.00, 144.50] |
| **BMI, kg/m2** | 25.88 [23.39, 29.65] |
| **Waist circumference, cm** | 82.00 [75.00, 91.00] |
| **Hip circumference, cm** | 101.45 [96.00, 108.15] |
| **Glucose, mmol/L** | 4.84 [4.53, 5.18] |
| **HbA1c, mmol/L** | 33.90 [31.70, 36.20] |
| **HDL-c, mmol/L** | 1.58 [1.34, 1.85] |
| **LDL-c, mmol/L** | 3.42 [2.90, 3.96] |
| **TG, mmol/L** | 1.30 [0.95, 1.81] |
| **Cholesterol, mmol/L** | 5.64 [5.01, 6.32] |
| **Testosterone, nmol/L** | 1.00 [0.71, 1.37] |
| **Estradiol, pmol/L** | 316.45 [228.50, 483.72] |
| **Age started hormone replacement therapy, y** | 48.00 [44.00, 51.00] |
| **HRT duration, y** | 5.00 [1.00, 10.00] |
| **Biological age, y** | 48.89 [44.16, 53.77] |
| **Biological age acceleration, y** | -5.53 [-7.68, -3.41] |

^a^ The description of continuous variables is represented by median and interquartile range, while the description of

categorical variables is represented by n (%).

^b^ Group differences were compared using Student t test, χ2 test, or Wilcoxon rank test.

Abbreviations: HRT, hormone replacement therapy; SBP, Systolic blood pressure; BMI, Body Mass Index; HbA1c, glycated haemoglobin A1c；HDL-c, High density lipoprotein cholesterol; LDL-c, High density lipoprotein cholesterol; TG, Triglycerides.

**eTable7. Association between HRT Initiation Age and Biological Age Acceleration**

| **Age Started HRT, y** | **BAA, mean (SD), y** | **HRT Duration, mean (SD), y** |
| --- | --- | --- |
| 36-40 | -5.07 (3.48) | 8.58 (7.43) |
| 41-45 | -5.33 (3.40) | 7.81 (6.59) |
| 46-50 | -5.44 (3.37) | 5.64 (5.09) |
| 51-55 | -5.99 (3.43) | 4.43 (4.15) |
| 56-60 | -6.21 (3.28) | 3.88 (3.34) |
| 61-65 | -4.96 (2.00) | 2.20 (2.59) |

**eTable 8. Sensitivity Analysis Using the Gompertz law-based biological age model**

| **Gender** | **Age Group,y** | **Estradiol, pmol/L** | | | **Testosterone, nmol/L** | | |
| --- | --- | --- | --- | --- | --- | --- | --- |
|  |  | **Est. ^a^** | **P** | **P. adj ^b^** | **Est.** | **P** | **P. adj** |
| **Female** |  |  |  |  |  |  |  |
|  | **41-45** | 0.04 (-0.05 - 0.13) | 0.35 | 0.38 | 0.05 (-0.03 - 0.14) | 0.21 | 0.25 |
|  | **46-50** | 0.07 (-0.02 - 0.16) | 0.11 | 0.14 | 0.09 (0.01 - 0.18) | 0.04 | 0.05 |
|  | **51-55** | 0.24 (0.11 - 0.37) | < 0.001 | < 0.001 | 0.19 (0.06 - 0.32) | 0.01 | 0.01 |
|  | **56-60** | 0.11 (-0.18 - 0.4) | 0.45 | 0.46 | 0.05 (-0.24 - 0.34) | 0.75 | 0.75 |
|  | **61-65** | -0.39 (-0.74 - -0.03) | 0.03 | 0.04 | 0.30 (-0.06 - 0.65) | 0.10 | 0.13 |
|  | **66-70** | -0.43 (-0.99 - 0.12) | 0.12 | 0.15 | 0.33 (-0.22 - 0.88) | 0.24 | 0.27 |
| **Male** |  |  |  |  |  |  |  |
|  | **41-45** | -0.16 (-0.43 - 0.11) | 0.26 | 0.31 | 0.65 (0.36 - 0.93) | < 0.001 | < 0.001 |
|  | **46-50** | 0.05 (-0.20 - 0.31) | 0.69 | 0.73 | 0.55 (0.28 - 0.82) | < 0.001 | < 0.001 |
|  | **51-55** | -0.03 (-0.30 - 0.23) | 0.80 | 0.82 | 0.35 (0.07 - 0.62) | 0.01 | 0.02 |
|  | **56-60** | 0.08 (-0.15 - 0.32) | 0.48 | 0.55 | 0.72 (0.47 - 0.96) | < 0.001 | < 0.001 |
|  | **61-65** | -0.16 (-0.37 - 0.05) | 0.14 | 0.17 | 0.68 (0.47 - 0.90) | < 0.001 | < 0.001 |
|  | **66-70** | 0.08 (-0.17 - 0.34) | 0.53 | 0.59 | 0.87 (0.60 - 1.13) | < 0.001 | < 0.001 |

^a^ The correlation and p-values between per SD sex hormone levels reduction and Biological Age Acceleration were determined using linear

regression analysis. Est. was presented using the coefficient (β) and 95% confidence interval (95%CI).

^b^ P. adj Adjusted factors include smoking status, alcohol consumption status BMI.

**
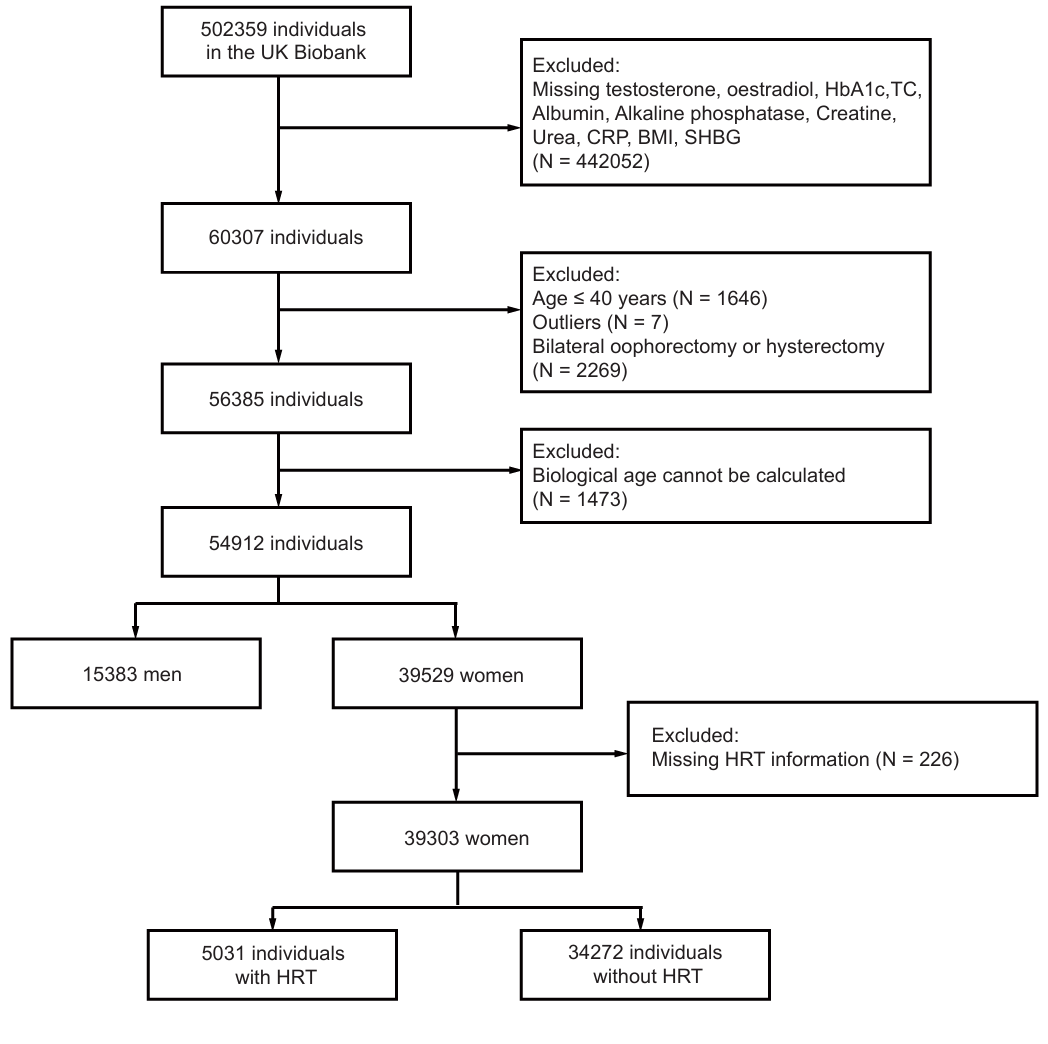
eFigure 1. Flow Diagram of the Analytic Sample**

Abbreviations: TC, total cholesterol; CRP, C reactive protein; SHBG, sex hormone binding globulin; BMI, Body Mass Index; HRT, hormone replacement therapy.


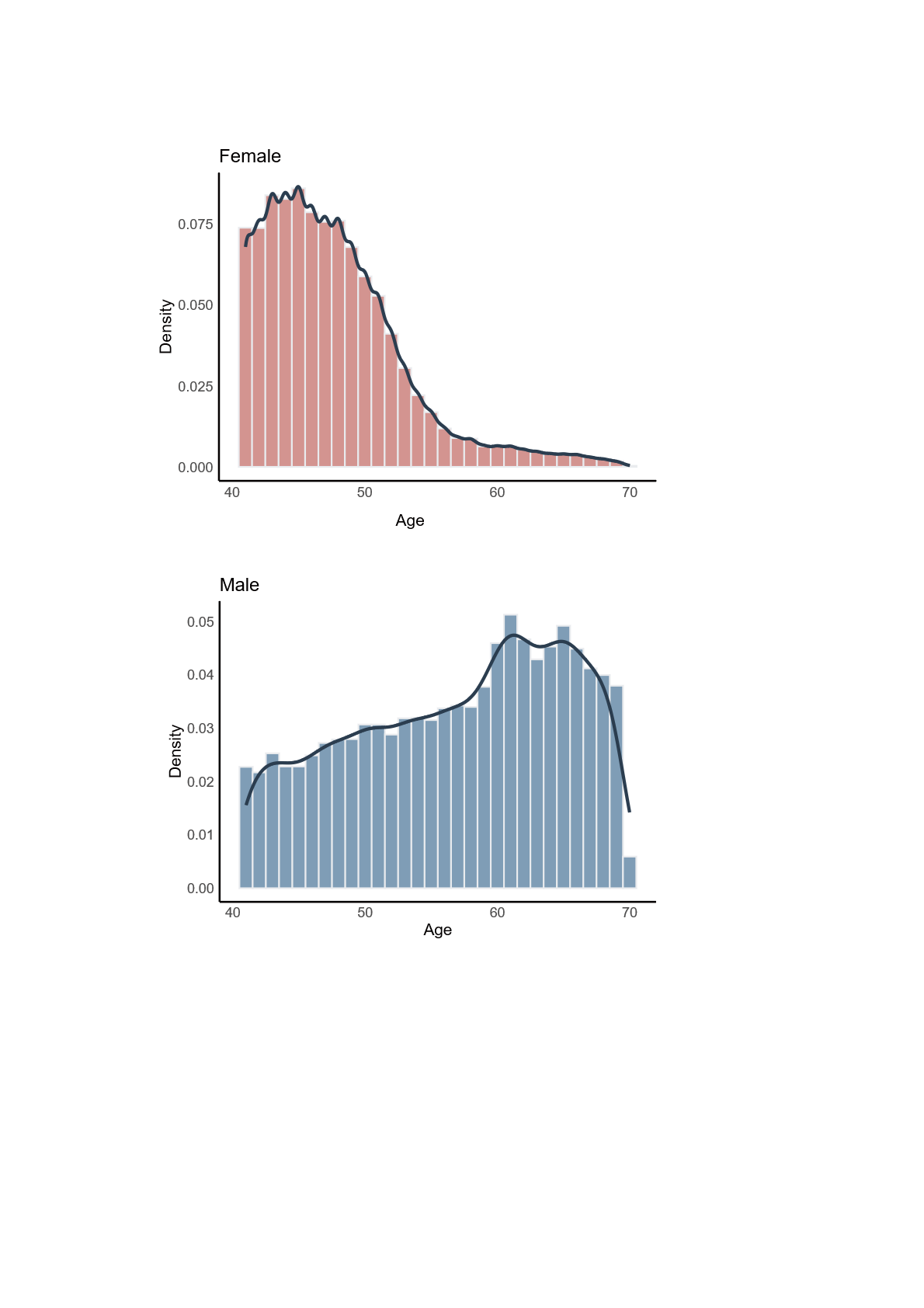


**eFigure 2. Age Distribution of Total Population**

**
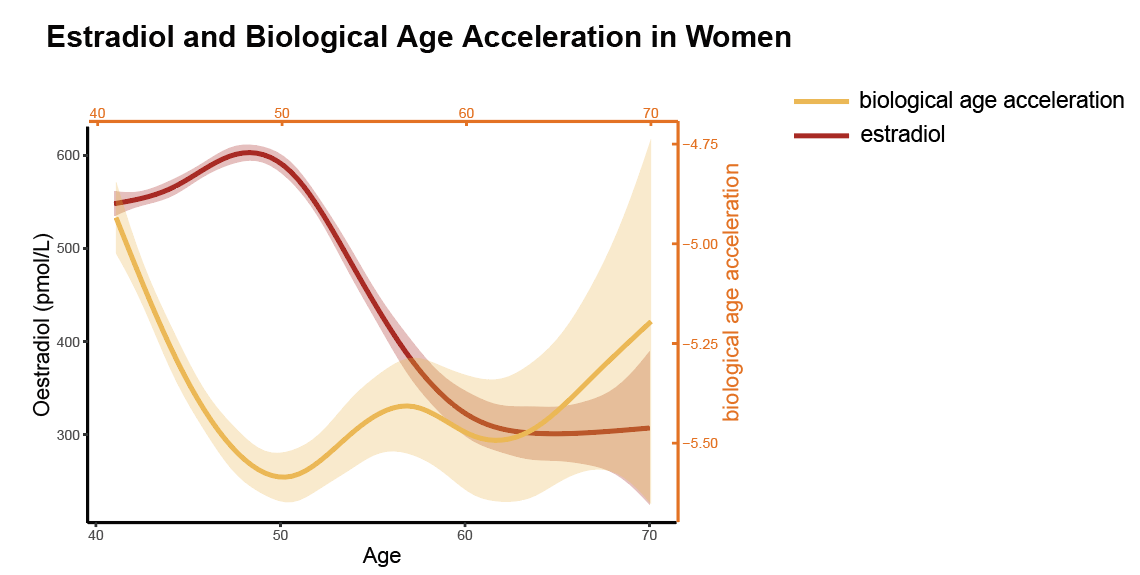
**
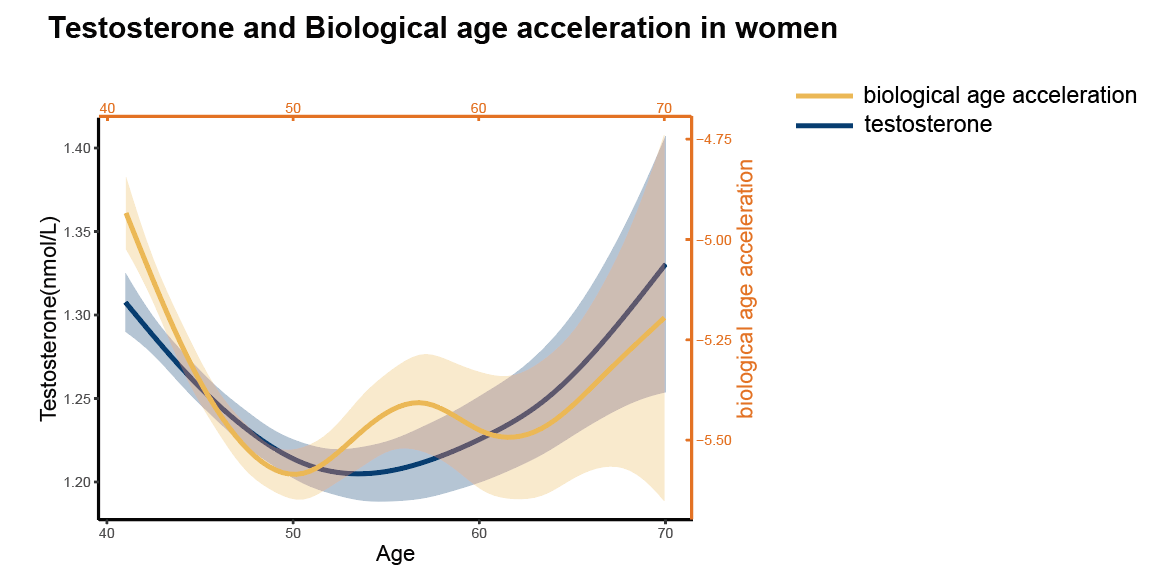


**eFigure 3. Trends in Sex Hormones and Biological Age Acceleration in Females**

**
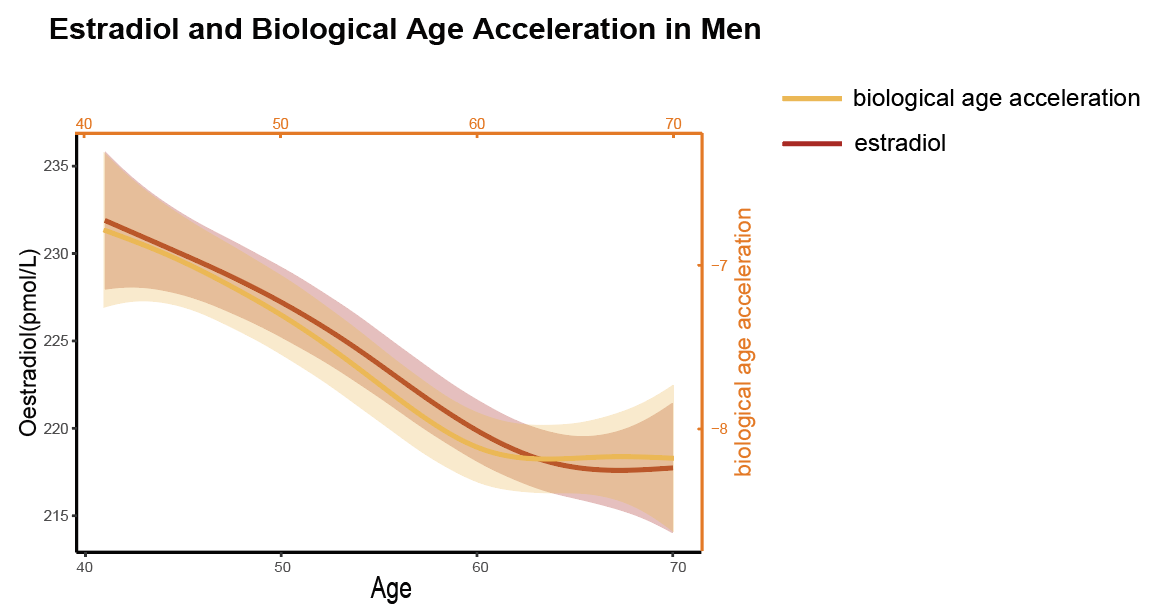
**
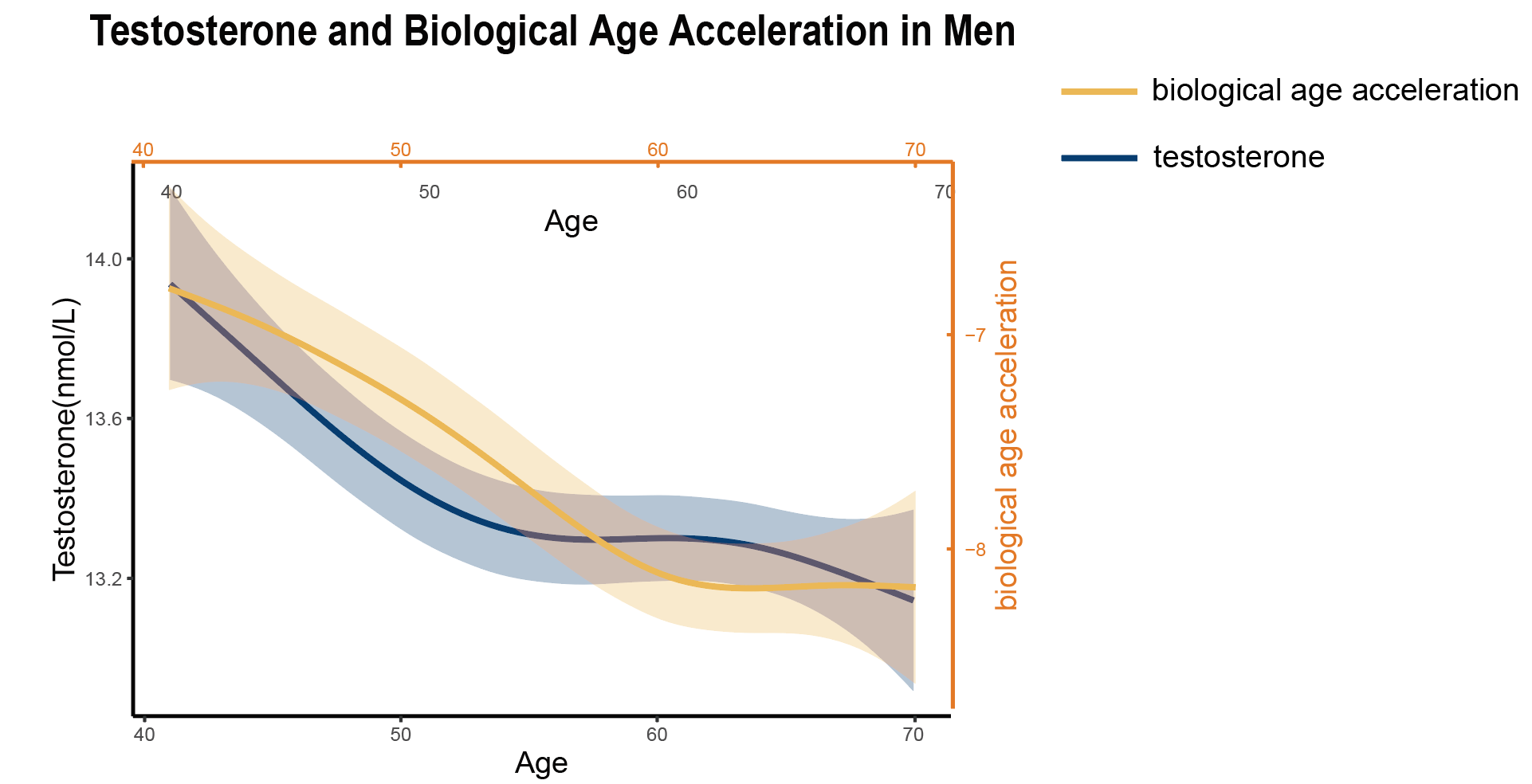
**eFigure 4. Trends in Sex Hormones and Biological Age Acceleration in Males**


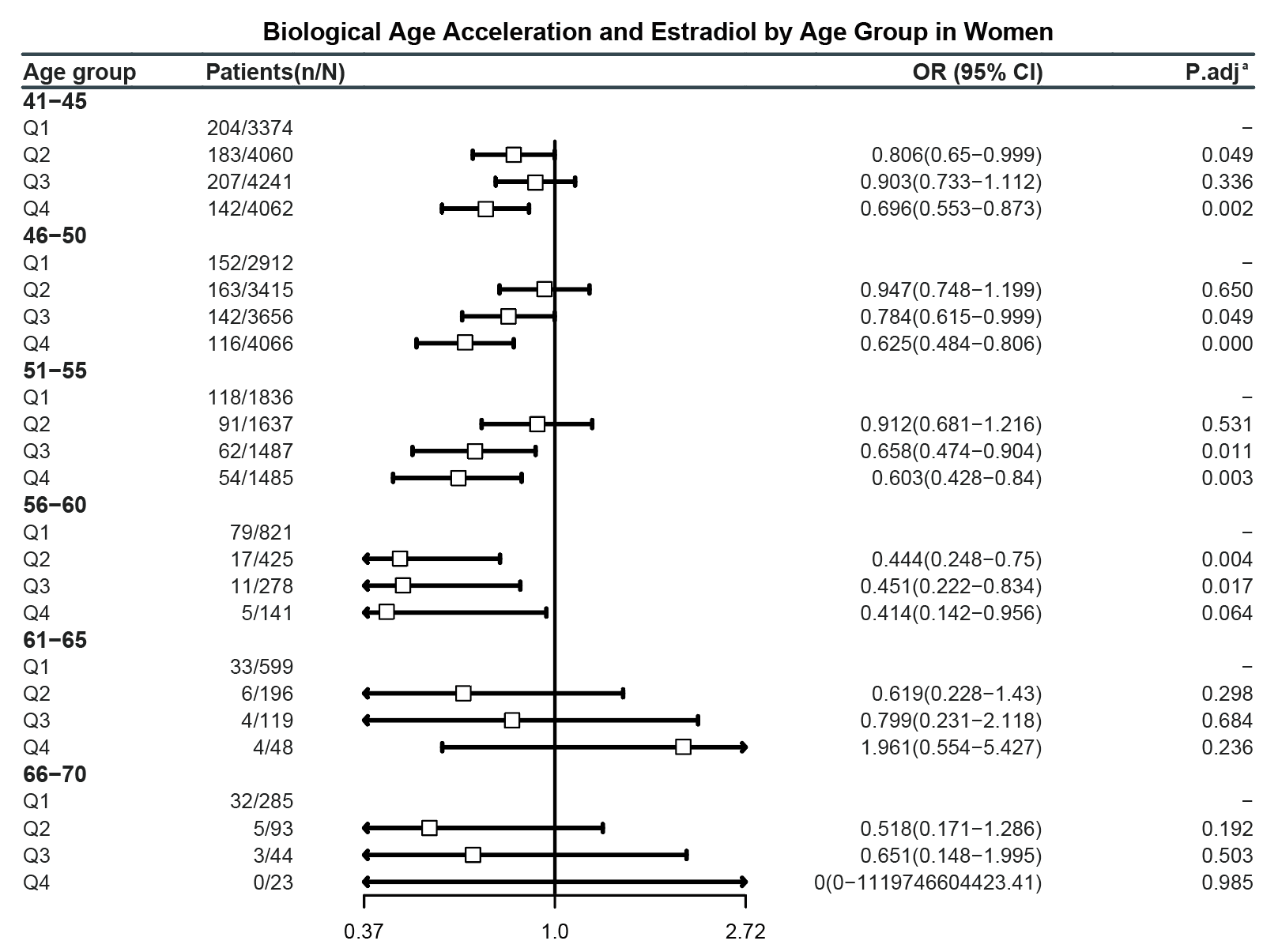

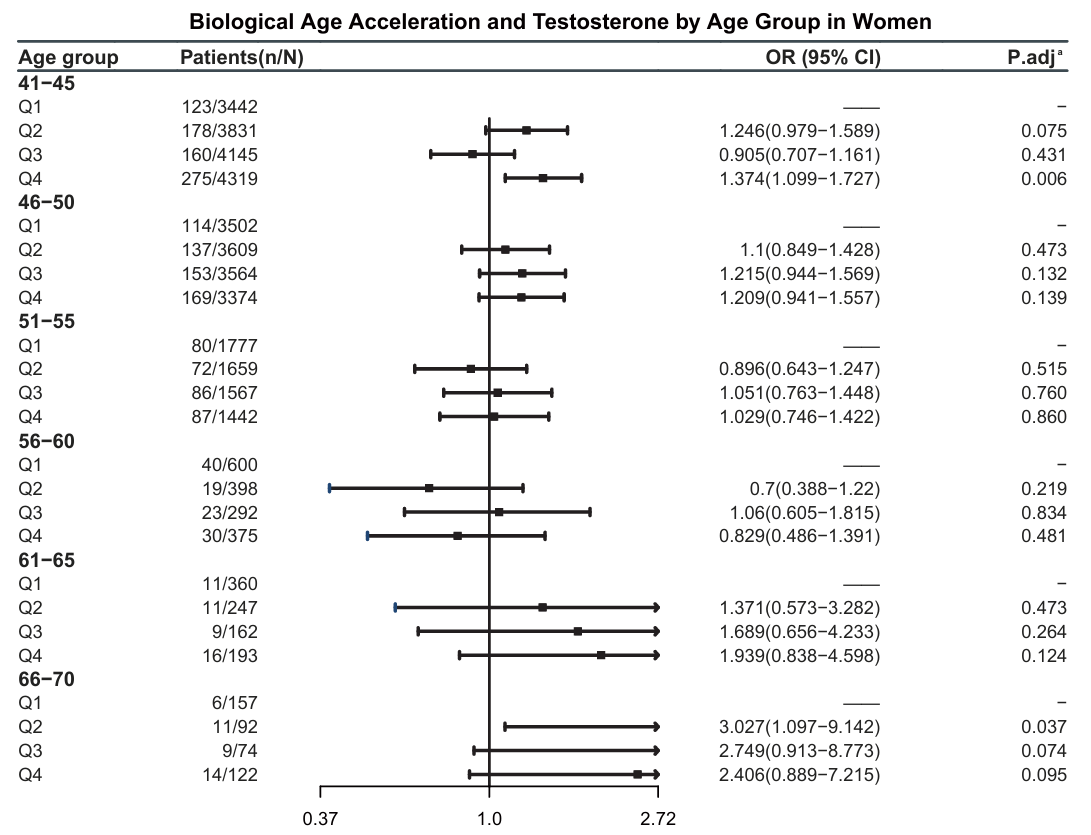
**eFigure 5. Association of Sex Hormones with Aging Acceleration in Females by Age Group**

Abbreviations: OR, odd ratio; CI, Confidence Interval; Aging Acceleration, biological age acceleration ≥ 0.

^a^ adjusted factors include Smoking Status, Drinking Status and BMI.

Estradiol quartile cutoffs (pmol/L) for females: 41-45y (Q1: 175-288, Q2: 288-425, Q3: 425-666, Q4: 666-7686); 46-50y (Q1: 175-293, Q2: 293-443, Q3: 443-720, Q4: 720-8911); 51-55y (Q1: 175-256, Q2: 256-382, Q3: 382-624, Q4: 624-6085); 56-60y (Q1: 175-212, Q2: 212-273, Q3: 273-410, Q4: 410-4496); 61-65y (Q1: 175-201, Q2: 201-235, Q3: 235-336, Q4: 336-2499); 66-70y (Q1: 175-195, Q2: 195-230, Q3: 230-332, Q4: 332-2838).

Testosterone quartile cutoffs (nmol/L) for females: 41-45y (Q1: 0.35-0.89, Q2: 0.89-1.20, Q3: 1.20-1.57, Q4: 1.57-17.57); 46-50y (Q1: 0.35-0.84, Q2: 0.84-1.14, Q3: 1.14-1.51, Q4: 1.51-13.24); 51-55y (Q1: 0.35-0.81, Q2: 0.81-1.11, Q3: 1.11-1.46, Q4: 1.46-16.72); 56-60y (Q1: 0.35-0.71, Q2: 0.71-1.02, Q3: 1.02-1.45, Q4: 1.45-29.42); 61-65y (Q1: 0.36-0.68, Q2: 0.68-0.99, Q3: 0.99-1.41, Q4: 1.41-31.42); 66-70y (Q1: 0.35-0.71, Q2: 0.71-1.08, Q3: 1.08-1.58, Q4: 1.58-22.79).


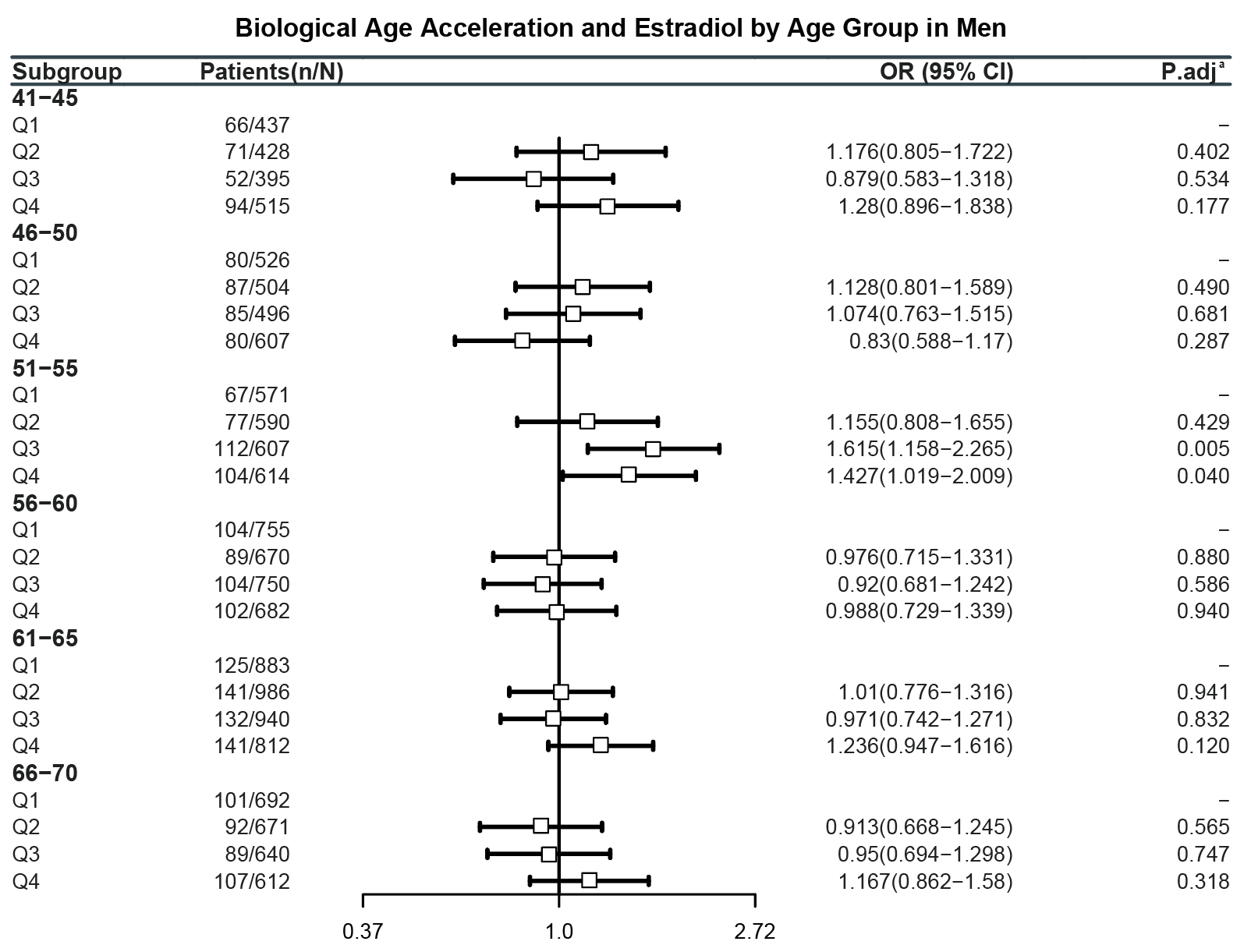

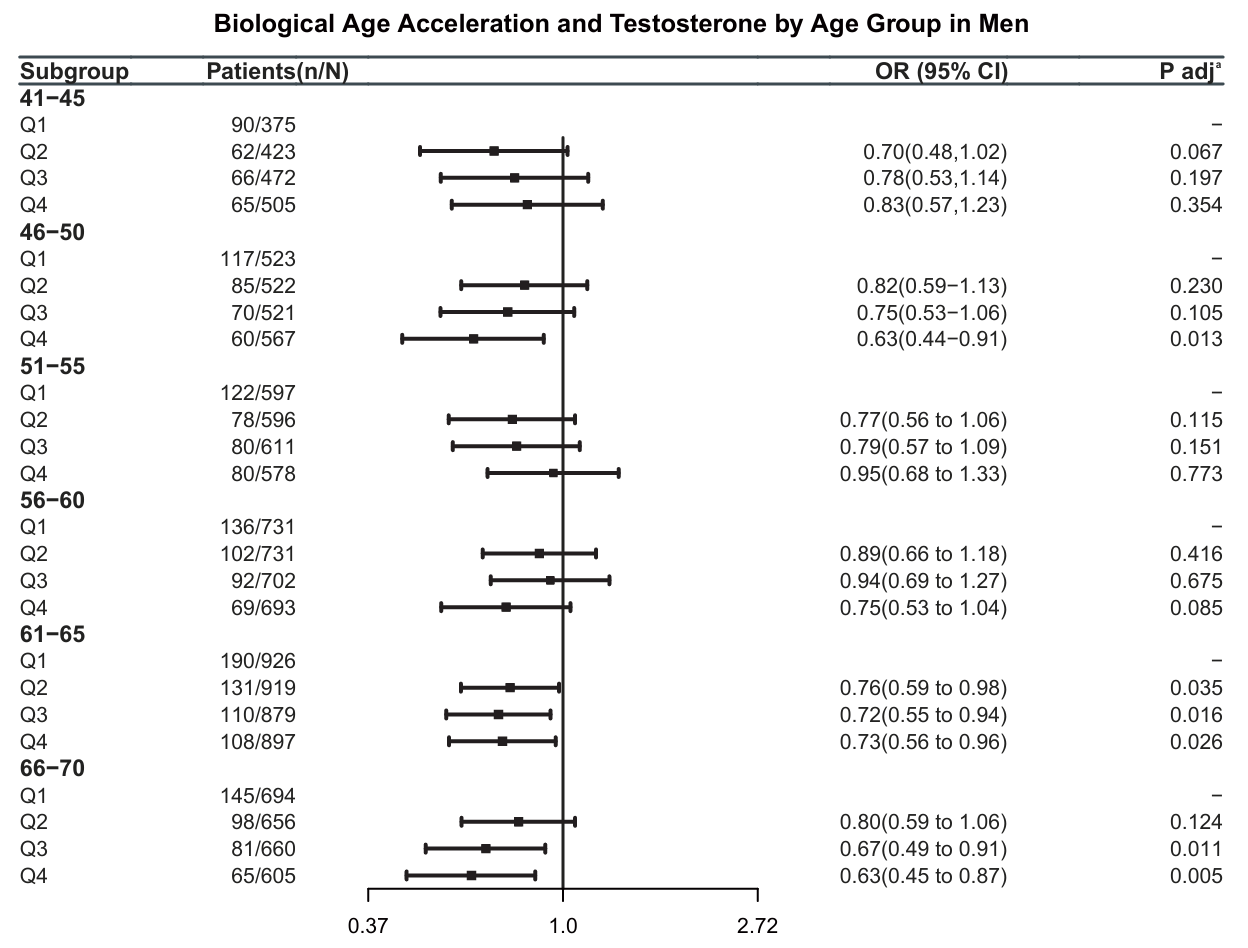
**eFigure 6. Association of Sex Hormones with Aging Acceleration in Males by Age Group**

Abbreviations: OR, odd ratio; CI, Confidence Interval; Aging Acceleration, biological age acceleration ≥ 0.

^a^ adjusted factors include Smoking Status, Drinking Status and BMI.

Estradiol quartile cutoffs (pmol/L) for males: 41-45y (Q1: 175-189, Q2: 189-205, Q3: 205-237, Q4: 237-1484); 46-50y (Q1: 175-189, Q2: 189-205, Q3: 205-238, Q4: 238-1029); 51-55y (Q1: 175-189, Q2: 189-205, Q3: 205-232, Q4: 232-1775); 56-60y (Q1: 175-188, Q2: 188-204, Q3: 204-230, Q4: 230-1230); 61-65y (Q1: 175-189, Q2: 189-203, Q3: 203-227, Q4: 227-1626); 66-70y (Q1: 175-188, Q2: 188-203, Q3: 203-228, Q4: 228-1756).

Testosterone quartile cutoffs (nmol/L) for males: 41-45y (Q1:0.74-10.92, Q2:10.92-13.39, Q3:13.39-16.20, Q4:16.20-48.46); 46-50y (Q1: 0.81-10.55, Q2: 10.55-12.98, Q3: 12.98-15.89, Q4: 15.89-52.43); 51-55y (Q1: 0.50-10.51, Q2: 10.51-12.89, Q3: 12.89-15.57, Q4: 15.57-53.14); 56-60y (Q1: 0.89-10.42, Q2: 10.42-12.79, Q3: 12.79-15.54, Q4: 15.54-43.33); 61-65y (Q1: 0.39-10.46, Q2: 10.46-12.79, Q3: 12.79-15.67, Q4: 15.67-47.35); 66-70y (Q1: 0.95-10.37, Q2: 10.37-12.71, Q3: 12.71-15.43, Q4: 15.43-51.17).
